# Multi-scale mechanical and acoustic characterization of low noise pavements

**DOI:** 10.1007/s11356-024-35198-2

**Published:** 2024-10-15

**Authors:** Lily. D. Poulikakos, Felix Schlatter, Liliane Huber, Peter Mikhailenko, Martin Arraigada, Michele Griffa, Christian Angst, Erik Bühlmann

**Affiliations:** 1 Empa, Swiss Federal Laboratories for Materials Science and Technology, Concrete and Asphalt Laboratory, Überlandstrasse 129, 8600 Dübendorf, Switzerland; 2 Grolimund+Partner AG, Waldeggstrasse 42a, CH-3097 Liebefeld-Bern, Switzerland; 3 IMP Bautest AG, Oberbuchsiten, Switzerland

**Keywords:** Pavement, Asphalt, Noise, Semi dense asphalt, Performance, Durability

## Abstract

Using data on in situ performance of low noise pavements, three well performing mixtures were selected: SDA 4, SDA 6, and SDA 8. These mixtures and the corresponding mastic (filler + bitumen + additive) were tested for their mechanical and acoustic performance in non-aged and aged states using a multi-scale approach (mm to km). Thereafter, an optimization protocol was applied to these mastics (mm-scale) and promising combinations were implemented at the mixture scale (cm-scale). As a result, the best performing mixture parameters were implemented in the m-scale slabs where the mechanical and acoustic performance were determined. The proposed work could uniquely combine knowledge from in situ long term and laboratory performance of low noise pavements. Using a traffic simulator and measurements before and after trafficking, the acoustic modeling showed that the modified mixtures’ noise levels were maintained after loading. This indicates that the modification assured acoustical integrity of the mixture after loading.

## Introduction

The World Health Organization (WHO) has reported that in the EU, the health of residents is affected by traffic noise listing it as the second largest environmental problem after air pollution (WHO 2011). Furthermore, in Switzerland, every seventh person is exposed to excessive road traffic noise (Federal Council 2017). The Swiss Federal Council therefore decided in 2017 to combat excessive road traffic noise more forcibly and effectively than before by taking measures at the source of noise which is the road/tire interaction point (Federal Council 2017). The implemented measures are speed reductions, traffic diversion, and low-noise pavements. Two factors influence the vehicle noise generation: engine noise and tire/road contact noise. As tire/road noise dominates at speeds above 20 km/h, low-noise pavements have become an effective noise abatement measure in Switzerland.

Comparative burden studies suggest that, after particulate matter, noise is the second major cause of Disability Adjusted Life Years (DALYs) lost (WHO 2017). This new health evidence highlights the urgency of adopting more stringent noise standards and implementing noise reduction technologies. One way to mitigate the exposure of residents to noise is to take advantage of the existing public spaces and transform the available surfaces of pavements into elements that foster comfort and health for urban residents. To this end, an effective noise abatement method has been the use of semi dense asphalt (SDA) pavements. Long-term in situ measurements of SDA pavements in Switzerland have shown that the acoustic performance of such pavements, although high after construction, can quickly diminish. Therefore, there is an urgent need to develop know-how on how to improve the acoustic and mechanical durability of such pavements.

Various parameters such as surface texture, porosity, interconnected voids, and aggregate size of the asphalt mixture contribute to the noise reduction character of a pavement (Tonin 2016). For example porous asphalt or open-graded asphalt can reduce the noise by 3–6 dB (Wehr et al. 2020). This is primarily because of the high porosity (around 25%) compared to conventional dense asphalt (4%) which enables the air to flow readily through the pavement, thus absorbing the noise (Wehr et al. 2020). Full-scale testing at a test track demonstrated that increased pavement surface porosity decreases noise levels almost at any frequency (Kuijpers et al. 2007). It is important, however, to balance porosity with mechanical durability of the pavement, because open-graded mixtures generally have a shorter lifetime compared to dense-graded pavements. For dense-graded mixtures, surface texture is the main parameter influencing the low noise property (Bennert et al. 2005; Liao et al. 2014). Low texture will reduce noise levels at lower frequencies (below 1600 Hz) (Kuijpers et al. 2007; Paje et al. 2008). Reduction of surface texture, however, comes at the cost of lowered tire friction; thus, one must find an optimum balance between noise and driving safety (Kuijpers et al. 2007). Research has shown effects related to vehicle and surroundings during driving, such as noise, rolling resistance, and tire wear, as a function of texture wavelength (Sandberg and Ejsmont 2002). It was shown that lower macro-texture has positive effects on road noise and the opposite effect on rolling resistance, which is a safety consideration. In Switzerland, SDA pavements have shown promising results (FOEN 2018) as discussed above.

SDA pavements are gap-graded mixtures. Their adoption has been one of the most effective noise abatement measures (Poulikakos et al. 2022). Optimizing their mechanical and acoustic durability makes such solutions cost effective during their service life which is considerably shorter than dense mixtures. The SDA mixtures have lower porosity than porous asphalt (PA) and theoretically combine the reduced noise properties with better mechanical durability in comparison to PA. Since 2010, over 1000 low-noise pavements were constructed in Switzerland. Thanks to the research efforts in recent years and many practical experiences, the asphalt mixtures have been improved mechanically as well as acoustically to a certain extent (FOEN 2018; Bühlmann et al. 2017). Nevertheless, questions remain about the durability of these pavements both mechanically and acoustically. The recently completed D-A-CH project of German speaking countries, ADURA (Akustische Dauerhaftigkeit lärmmindernder dichter oder semi-dichter Asphaltdeckschichten), shows that the acoustic durability of SDA can be influenced by the amount and choice of binder (Wehr et al. 2020). The project, however, does not provide specific solutions for increasing the durability of SDA. Since the lifecycle cost analysis of SDA are largely influenced by their service life, improving the acoustic and mechanical durability of SDA remains a key priority in order to holistically assess the effectiveness of this pavement type as a noise abatement measure.

## Research goals

The current research aimed to improve the mechanical and acoustic durability of various low noise pavements. Based on the current state of in situ performance of low noise pavements, using data from 200 test sections, three acoustically well performing mixture types were selected: SDA 4, SDA 6, and SDA 8. These mixtures were tested for their mechanical and acoustic performance in non-aged and aged states using a multi-scale approach. Thereafter, an optimization protocol was applied to these mixtures. Thereby, the best performing mixtures were selected, and optimization parameters identified. The project aimed to uniquely combine knowledge from in situ long-term performance and laboratory performance tests of low noise pavements.

## In situ acoustic data

**Fig. 1:**
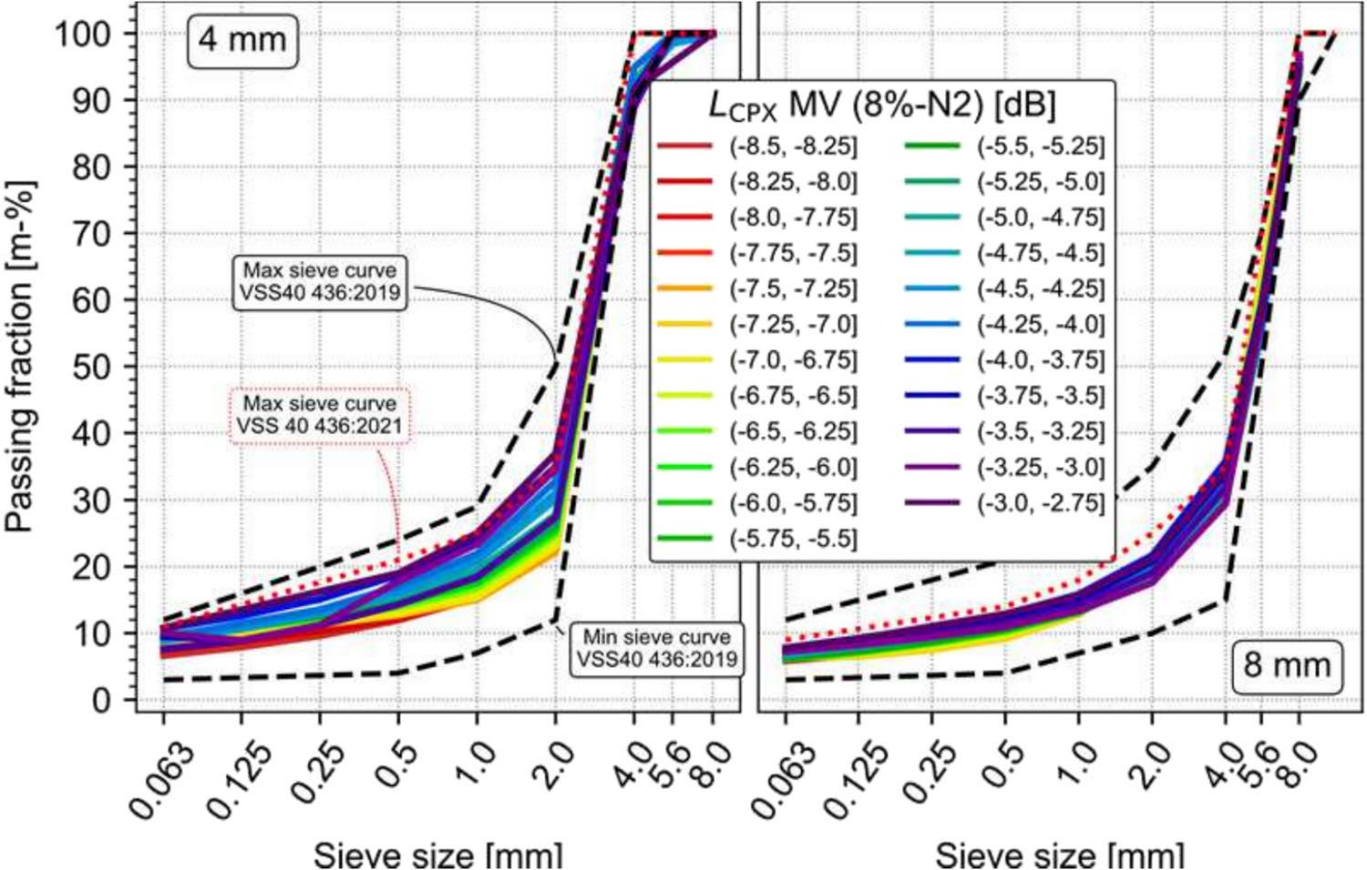
In situ (initial) acoustic performance of SDA 4 (left) and SDA 8 (right) mixtures, along with the mixture’s grading curve. Each curve is color-coded based upon the acoustic performance index given in parenthesis. Dotted black lines indicates the upper and lower bounds as defined by the SN VSS40 436:2019, whereas the red dotted lines indicate the upper bounds of the current valid standard SN VSS40 436:2021

In Switzerland, since 2012, data regarding acoustic performance of SDA 4 (semi-dense asphalt with 4 mm maximum aggregate size) and SDA 8 (semi-dense asphalt with 8 mm maximum aggregate size) pavements comprising 2500 CPX (close proximity acoustic) measurements for over 1000 SDA sections has been collected. Data on 200 sections including the construction data (it is required to report certain mixture data for SDA construction such as bitumen content, gradation, Marshall voids, voids of drill cores and layer thickness) as well as acoustic performance data from the CPX measurements required for low-noise pavements. As part of analyzing the construction data, drill cores were analyzed from 775 different mixtures. This data allows to relate the acoustic performance after their service years of such mixtures with their aggregate gradation. Figure 1 shows the relation between gradation and acoustic performance for SDA 4 and SDA 8 mixtures, with warmer colors indicating better acoustic performance. Here all the mixtures are characterized with respect to their (initial) acoustic performance. This data allows to make recommendations for an optimal recipe from an acoustic point of view. The goal was to reduce the voids content as much as possible to improve mechanical performance but to have acoustically active pores, which are pores that are connected to the surface. Figure 1 further demonstrates that although all the mixtures examined fall within the requirements of the old Swiss standard (VSS 40 436:2019, black dashed lines), they can have very different acoustical performance in situ. Furthermore, it is apparent that getting closer to the lower part of the curve can be acoustically beneficial. The new standard (VSS 40 436:2021) therefore partially addresses this issue and has somewhat restricted the maximum sieve curve. In order to strike a balance between acoustic and mechanical performance, a voids content of 15% was recommended for this project.

## Materials

Using the aggregate gradation discussed above and aiming for 15% air voids, the baseline mixtures were prepared. The aggregates were limestone from Massongex, Switzerland (FAMSA), complying with the performance requirements for SDA by Swiss standards (SN 640 436) (2013). Three types of filler were used: a weak limestone filler (lower bound of standard), a filler (Zeofill) with a strong stiffening effect (upper bound of standard), and a special type of filler (Zeobit) with a very strong stiffening effect. Calcium hydroxide or lime was used as an additive to enhance the water sensitivity of the mixtures (Gorkem and Sengoz 2009).

Two types of bitumen were used for this project. PmB 45/80–80 (CH-E) (designated as B1) is highly polymer-modified with penetration of 53 (0.01 mm) and softening point of 92.5 °C and PmB 45/80–65 (CH-E) (designated as B2) is also polymer-modified with penetration of 66 (0.01 mm) and softening point of 84 °C.

**Fig. 2:**
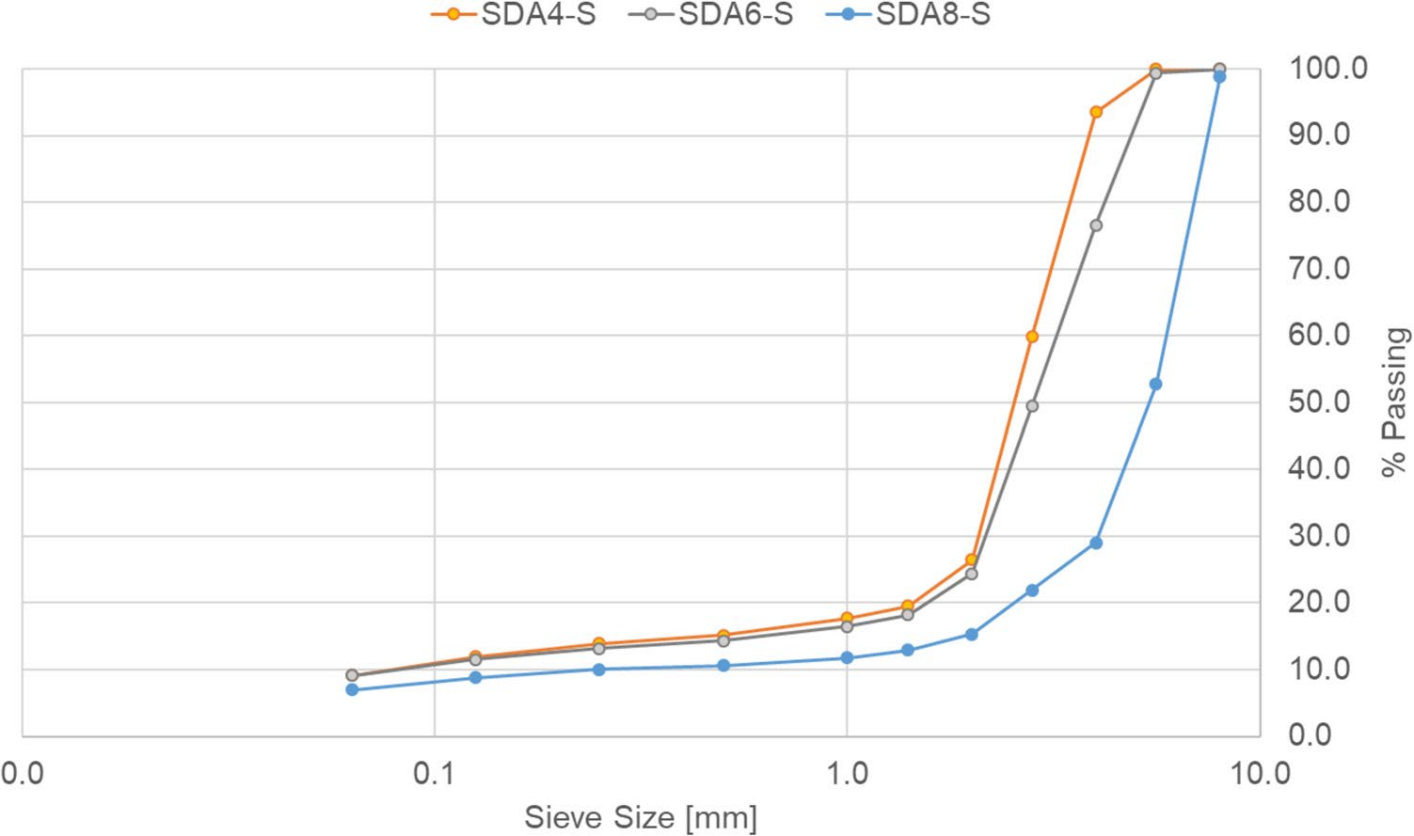
Gradation of SDA4-S, SDA6-S, and SDA8-S

Table 1 and Fig. 2 shows the gradation and properties of the three benchmark mixtures namely, SDA 4-S, SDA 6-S, and SDA 8-S mixtures as well as the corresponding modified mixtures namely SDA 4-M1, SDA 4-M2, and SDA 4-M3, where S stands for standard or reference mixture and M for modified mixture. The materials used are shown in Table 2.

**Table 1:** Mixture properties

Sieve size	Mixture designation and passing fraction (Mass-%)					
[mm]	SDA 4-S	SDA 6-S	SDA 8-S	SDA 4-M1	SDA 4-M2	SDA 4-M3
8.0	100	100	98.9	100	99.9	100
5.6	100	99.4	52.7	99.9	99.8	99.9
4.0	93.5	76.6	29	95.3	94.5	94.2
2.8	59.8	49.4	21.9	64.6	63.8	60.9
2.0	26.5	24.4	15.3	28.6	27.9	27.7
1.4	19.5	18.2	12.9	19.9	19.0	19.1
1.0	17.7	16.5	11.8	17.5	17.0	16.9
0.500	15.2	14.3	10.6	15.1	14.4	14.1
0.250	13.9	13.2	10	13.9	13.2	13.2
0.125	11.9	11.5	8.8	12.9	12.2	12.2
0.063	9.1	9.1	7	9.7	8.8	9.1
Binder content (Mass-%)	5.4	5.8	5.3	6.2	5.8	6
Max density (g/m^3^)	2.48	2.47	2.46	2.45	2.47	2.45
Bulk density (g/m^3^)	2.1	2.10	2.07	2.09	2.09	2.13
Voids content (Vol%)	16.3	14.9	15.8	14.7	15.5	13.2
VFB Voids filled with bitumen (Vol-%)	*	44.4	40.4	46.1	43.2	48.3
VMA Voids in asphalt mixture (Vol-%)	*	26.8	26.5	27.2	27.2	*
Richness modulus (-)	*	3.6	3.5	3.8	3.6	3.7

*Data not available

**Table 2:** Materials and mixture designation

Designation	Description
SDA 4-S	Benchmark SDA 4 mixture with PmB45/80-65E (B2), Zeofill filler (ZF)
SDA 6-S	Benchmark SDA 6 mixture with PmB45/80-65E (B2), Zeofill filler (ZF)
SDA 8-S	Benchmark SDA 8 mixture with PmB45/80-65E (B2), Zeofill filler (ZF)
SDA 4-M1	Modified SDA 4 mixture with highly modified bitumen (B1), Zeobit filler (ZB)
SDA 4-M2	Modified SDA 4 mixture with highly modified bitumen (B1). limestone filler (L)
SDA 4-M3	Modified SDA 4 mixture with highly modified bitumen (B1), Zeobit filler (ZB), calcium hydroxide (hydrated lime) as additive
SDA 8-M3	Modified SDA 8 mixture with highly modified bitumen (B1), Zeobit filler (ZB), calcium hydroxide (hydrated lime) as additive

## Experimental methods

A multi-scale experimental method was utilized to investigate the performance of these mixtures more efficiently. First various binder, filler and additives were investigated in the mastic (i.e., mm) scale. From the gained new knowledge, mixtures were fabricated for the cm-scale investigations. From the cm-scale results, additional mixtures were further selected for the m-scale laboratory investigations.

### Mastic tests (mm-scale)

To produce a mastic mixture, the received fillers were sieved down to 0.125 mm and dried for 2 days in the oven. Firstly, the base bitumen was heated to reach a molten state at 145 °C. Thereafter, the different fillers and additives, also at 145 °C, were gradually introduced into the binder using a low-speed mixer at 145 °C. Next the blend was heated to 180 °C and homogenized again, before the samples were molded into the sample holders for the DSR measurements and the containers for the ageing.

The mastic was aged using the PAV (pressure ageing vessel) method (SN 670 558/EN 14769−2012). Then 50 g of mastic per container was prepared and put into the vessel for 20 h at 100 °C and 21 bar pressure. Afterwards, the samples were homogenized and allowed to cool. The procedure was repeated for the second ageing step (2PAV). At this point, samples were also prepared for the rheometer measurements (DSR) after one ageing step. After the second ageing step, the containers were again combined, homogenized, and allowed to cool. As preparation for the DSR measurements the sample was again heated to 180 °C and homogenized before it was poured into the prepared molds for the DSR.

The normally performed Rolling Thin Film Oven Test (RTFOT) (SN EN 12607–1) that simulates short-term ageing was not performed, as this is not possible with a mastic mixture. However, as the aim was to see whether the different fillers influence the ageing effect of the mixture, the short-term ageing was deemed not relevant.

### Mastic rheological testing using the DSR

This European standard (EN 14770−2012) specifies a method to measure the rheological properties of bituminous binders using a dynamic shear rheometer (DSR). The complex shear modulus and phase angle is determined at certain frequencies and temperatures when tested in oscillatory shear. From the test, the norm of the complex shear modulus, IG*I, and its phase angle, δ, are calculated. The tests were performed at frequencies of 0.01 to 100 rad/s and temperatures of − 10–40 °C in 10 °C steps. In addition, a temperature sweep from 30 to 90 °C with a frequency of 1.59 Hz was performed with an increment of 10 °C. Before the frequency sweep tests, the linear viscoelastic (LVE) range of different binders was determined through strain amplitude sweep tests to guarantee that the frequency sweep tests were undertaken within the binder’s LVE response region. The low temperature range was performed with a plate-plate geometry of 8-mm diameter and a gap width of 2 mm, while the higher temperatures were measured with the 25-mm plate-plate geometry with a gap width of 1 mm. The results of the overlap of the two geometries agreed and were confirmed within expected uncertainties. Furthermore, at least two measurements with the same geometry had to be within 5% to be accepted as reasonable.

### Mixture preparation and compaction

The aggregates and bitumen were heated to the mixing temperature of 170 °C in the oven. The compaction was done at 155 °C using the Marshall compaction with 50 blows per side or a gyratory compactor to the required height. The samples that were produced for water sensitivity tests were under compacted to 50 gyrations as required by the standard.

Once the mixture was prepared, it was spread to a thickness of 3–4 cm on a metal sheet and placed in the oven at 155 °C for 2 h. This aging procedure replicates the state of the mixture after manufacturing in the asphalt mixing plant and until it is laid. The mixing plants in Switzerland are normally close to the job sites.

### Mixture tests (cm-scale)

#### Indirect tensile strength

Six cylindrical specimens were compacted for each type of mixture using the gyratory compactor to the target air voids listed in Table 1. According to EN 12697–12 (2008), a “dry” and “wet” subset was produced. The three dry samples were stored at room temperature (20–25 °C) while the wet samples were water conditioned at 40 °C for 72 h. After conditioning, the indirect tensile strength (ITS) test was performed at 25 °C with a displacement rate of 50 ± 2 mm/min (EN 12697–23 (2017)). ITS was calculated in accordance with Eq. (1) using the peak load *P* (N) and diameter *d* (mm) and height *h* (mm).

1 $$ITS=\frac{2.P}{\uppi .d.h}$$

Three replicates were tested for each mixture type and average values were calculated.

#### Water sensitivity

The moisture sensitivity of asphalt mixtures was calculated as the ITS ratio (ITSR) in percent as follows:

2 $$ITSR\left(\text{\%}\right)=\frac{ITS_{wet}}{ITS_{dry}}\cdot100\;\lbrack\text{\%}\rbrack$$

where ITS_wet_ is the average ITS of the wet group (kPa) and ITS_dry_ is the average ITS of the dry group (kPa).

#### French rutting test

Two slabs per mixture type with dimension 500 mm (length) × 180 mm (width) × 50 mm (thickness) were compacted for the rutting test. The European standard EN 12697–22 (2003), Procedure B, prescribes the test to be done in air at 60 °C, using a pneumatic wheel loaded to 700 ± 10 N that travels a distance of 230 ± 10 mm at a frequency of 26.5 ± 1 cycles per minute. The rut depth is measured after a selected number of passes.

#### Cyclic compression test

The cyclic compression test was used to investigate creep performance of the mixtures following the European Standards EN 12697–25 (2016) method A2 which allows determination of permanent deformation at high temperature (50 °C). A haversine loading cycle was applied for 1.7 s with a rest period of 1.5 s. A chamber was used to condition the samples (100-mm diameter and 60-mm height, surfaces polished plane parallel) at 50 °C for at least 4 h before the tests. The compressive loading is applied through two loading plates at top and bottom of specimen, with upper and lower stresses of 0.35 MPa and 0.025 MPa, respectively. Two sets of specimens were used for each test. From the experiments, a creep curve can be realized showing the cumulative axial strain, in %, as a function of the number of loading cycles. The creep curve typically shows three stages but depending on the material some of these stages might be absent. Stage 1 is exemplified by a decrease in the slope; in stage 2, the slope remains constant and from this stage the creep rate, fc, can be calculated; in stage 3, the slope increases and the turning point can be determined in this stage. The cumulative axial permanent strain ε_n_, in percent (%), can be calculated using Eq. (3). The test is ended when 10,000 cycles are reached or the cumulative axial strain ε_n_ has reached 40%.

3 $${\epsilon }_{n}=\frac{{u}_{n}}{{t}_{i}}.100$$

where ε_*n*_ is the cumulative axial strain of the test specimen after *n* loading cycles, in percent (%); *u*_*n*_ is the cumulative permanent deformation of the test specimen after *n* loading cycles; and *t*_*i*_ is the initial thickness of the test specimen in mm.

#### Semi-circular bend test

The semi-circular bending (SCB) test was used to determine the fracture resistance and it has been reported to be a useful testing method for mixture design and quality control (Molenaar et al. 2002; Mikhailenko et al. 2020).

The test was performed using a loading strip moving at a rate of 5 mm/min on 100-mm diameter specimens with a 3.5 by 10 mm notch at 0 °C as prescribed by the European standard EN 12697–44 (2019). Although two replicates were required, here four were tested.

#### Stiffness modulus

The stiffness modulus was determined using the Cyclic Indirect Tensile Test (CIT-CY) according to the European standard EN 12697–26 (2012). The tests were performed on four samples per compacted mixture under sinusoidal loading. The procedure is used to rank bituminous mixtures on the basis of complex modulus. In addition, it is used for measuring the mechanical response of the mixtures at a range of temperatures and frequencies.

Cylindrical samples were compacted using the gyratory compactor to target voids, and cut to 100 mm in diameter and 40 mm in height. The test is performed in the linear range of the material, under repeated loads. The linear-elastic range was determined at the different frequencies and temperatures by increasing the load in small increments. The tests were conducted at frequencies of 0.1, 1, and 10 Hz and temperatures of 10, 15, and 20 °C. The amplitudes of the stress and strain are measured, together with the phase difference between stress and strain.

#### Fatigue

Fatigue life is an important parameter in performance of materials as most materials undergo a gradual deterioration under repeated stresses that are much smaller than the ultimate strength of the material. Therefore, knowledge of fatigue behavior of asphalt concrete materials is imperative for material evaluation. The fatigue tests were performed as per EN 12697–24 (2007). The specimen is loaded sinusoidally at the imposed displacement amplitude until it reaches a certain failure criterion. The goal is to measure the number of cycles *N*_*i*_ at failure in a Wöhler type of diagram. At least nine specimens are tested for the IT-CY fatigue test. The strain ε_*i*_ is chosen on a logarithmic scale; so that three levels of deformation are tested, with normally six specimens at each level.

On the basis of the results, different fatigue life values, *N*_*i*_, are determined for different maximum applied initial strain ε_*i*_. A linear regression is plotted between the decimal logarithms of *N*_*i*_ and the decimal logarithms of ε_*i*_ as follows:

4 $$\mathrm{Log}\;\left(N\right)=\mathrm a+(1/\mathrm b)\cdot\log(\varepsilon)$$

where ε is the strain; *a* is the ordinate of the fatigue line; 1/*b* is the slope of the fatigue line. *R*^2^ is the linear correlation coefficient (log(*N*_*i*_), log(ε_*i*_)). A high *R*^2^ value is an indication of the goodness of the fatigue curve. In this study, all fatigue lines have been calculated using decimal logarithm (log) as in Eq. (4). The initial complex stiffness modulus *E* × 100 (or S mix, 0 as defined by the standard) is measured after 100 load applications. The conventional failure criteria (at constant displacement amplitude) defined in the standard as fatigue life is the number of load applications, *N*_f/50_, at which point the modulus has decreased to half its initial value. ε_6_ is used as a measure of fatigue and is the strain at one million cycles.

#### Particle loss

For particle loss measurements, the modified micro-Deval (MDI) tests was used. In the case of MDI, the drum described in EN 1097–1 (2011) is used that is considerably smaller (inside diameter 200 mm and internal length of 154 mm) than the drum used in the alternate particle loss Cantabro test. Additionally, the particle loss is simulated by the contact with the obstacle in the drum and not a ball. The mixture is aged at 85 °C for 120 h. Thereafter, the Marshall samples are under compacted at 2 × 40 blows to weaken the sample in a targeted manner and water conditioned according to EN 12697–12 (2008) before the test. The sample is placed in the drum and the drum in rotated at a speed of (100 ± 5) min^–1^ for (12 000 ± 10) revolutions. Thereafter, the mass loss is measured.

5 $$MV=\frac{(M1-M2)}{M1}.100$$

where MV is the mass loss in %, and M1 and M2 are the mass before and after testing.

#### Trafficking with the MMLS3

The Mobile Model Load Simulator MMLS3 is a laboratory-sized, accelerated traffic device, with four pneumatic tires that can load the surface of a pavement over a length of about 1.2 m, simulating the real vehicular loading effect on a structure. This type of test uses rolling tires to induce damage in scaled pavements or asphalt slabs, in a similar way that traffic deteriorates roads (Arraigada et al. 2014). Depending on the conditioning temperature, it can be used to study the rutting or cracking performance of a mixture. Therefore, with this tool, it was possible to upscale the laboratory results obtained on cylindrical specimens and validate them using repetitive rolling tires and slab specimens. Specifically, this method allowed to obtain information about the mixtures behavior in the wheel path (trafficked) and outside the wheel path (not trafficked). To prepare the slabs, the mixtures were poured in a frame and compacted with a steel roller compactor. The pneumatic tires of the device were set with a pressure of 6 bar and a load of 2.1 kN. Their speed was fixed at 4.5 km/h. The experimental setup allows only one tire to be in contact with the pavement at a time. These conditions corresponds to 60 tires passes per minute, which is equivalent to 1-Hz loading frequency, i.e., about 3600 unidirectional tire passes per hour.

For each material, two slabs with dimensions 1600 × 430 × 50 mm were produced making a total of eight slabs for testing. The experiments consisted of laying the slabs on top of a rigid concrete plate and loading them with the MMLS3 in a temperature chamber at 40 °C to study the rutting behavior, whereas other measurements to assess the acoustic, texture, and air resistivity performance were carried out at regular accumulated loading intervals for each mixture, as described in the next sections. Further, at the end of the loading phase, cores were taken to study the internal structure by X-ray tomography.

#### Rutting performance

Permanent deformation of the slabs was carried out using a profilometer, which picks up the slabs surface shape, perpendicular to the loading direction. Due to the permanent deformation caused by the repetitive and channelized tire rolling, the profiles obtained show the progression of the rut shape with the accumulation of load. For each profile measurement carried out at regular loading intervals, it is possible to obtain the rutting (rut depth) as the difference between the highest and lowest point in the profiles. As a result, a rut depth vs. MMLS3 loading curve is obtained, showing the susceptibility of the material to permanent deformation.

#### Acoustic tests on mixtures

In order to characterize the mixtures in terms of the (initial) acoustic performance, several tests were planned to describe the mixtures and to differentiate the mixtures and the optimized slabs. All acoustic characterizations were performed on the large slabs produced for the MMLS3 tests as the compaction with a roller compactor can represent the field compaction better than the gyratory compaction for the cylindrical laboratory specimens. This allowed to obtain data in the wheel path as well as outside of the wheel path thereby investigating the effect of loading cycles on the development of the material.

#### Surface texture

The surface texture of the MMLS3 tested slabs was evaluated using an Ames Engineering 9400HD 3D laser scanner. The scanning was performed by placing the device horizontally on the slabs. The scanned region had an area of 102 × 50 mm^2^ in the direction of the wheel path, in and out of the wheel path. The vertical, longitudinal, and width resolutions were 0.005 mm, 0.006 mm, and 0.2 mm respectively. These scans were used to calculate the texture level (*L*_TX,λ,_ expressed in dB) as a function of center wavelength of the 1/3 octave band (Paje et al. 2008), λ, by taking the 1/3 octave band power spectral density *Z*_p,λ_ of the surface profile amplitude along each scan line and by using Eq. (6) derived from ISO 13473–4:

6 $${{\varvec{L}}}_{\mathbf{T}\mathbf{X},{\varvec{\uplambda}}}=10\cdot \mathbf{l}\mathbf{o}\mathbf{g}\left(\frac{{{\varvec{Z}}}_{\mathbf{p},{\varvec{\uplambda}}}\cdot 0.232/{\varvec{\uplambda}}}{{{\varvec{a}}}_{\mathbf{r}\mathbf{e}\mathbf{f}}^{2}}\right)$$

where 0.232/λ is the corresponding bandwidth; and *a*_ref_ is the reference value of the surface profile amplitude (10 − 6 m according to ISO 13473–4).

#### X-ray tomography

X-ray tomography was used for assessing the fraction of a specimen’s volume constituted by interconnected pore regions. It was performed on cylindrical, cored specimens (about 50 mm in diameter, about 50 mm in height) with an in-house developed tomograph located at Empa’s Center for X-ray Analytics. The detailed description of the instrument as well as the measurement settings are provided in Appendix 1 at the article’s end.

We remark here that, for the given specimen size, the X-ray source needed to be operated at 180 kV and 500 μA, to achieve a sufficient signal-to-noise ratio in the acquired radiographs. The voxel size of each tomogram was $\widetilde{p}\cong$ 0.03 mm, while its effective spatial resolution was approximately at the scale of double that value (0.06 mm), as explained in the Appendix.

#### Acoustic absorption

The sound absorption properties of a road surface are highly frequency-specific and depend on its layer thickness, porosity, pore shape, their degree of interconnection, and the specific flow resistance. The sound absorption properties of a road surface can have a great influence on the extent of the horn effect and on sound propagation. Their metrological determination is of great importance in the closer analysis of the acoustic effectiveness of semi-dense and porous pavements.

The sound absorption properties are measured in situ using the PU ((Sound-) pressure, P, and -velocity U) method. In this measurement method, a sound signal is emitted with a loudspeaker onto the test surface. In addition to the sound pressure, the sound velocity is also recorded. From this, the impulse response of the emitted signal and its reflection are calculated and the sound absorption coefficient of the test surface is determined. Compared to the determination of the absorption coefficient with the impedance tube method, the PU method can be used to determine the sound absorption coefficient for a wider range of the noise spectrum. Furthermore, it is possible to determine relatively low sound absorption properties in situ with high accuracy.

The sound-absorbing properties of a road surface are best when the highest possible sound absorption coefficient is realized in the frequency range in which the most sound energy is generated when vehicle tires roll over the road surface. This is typically in the middle frequency range between 800 and 1250 Hz.

Normally, sound absorption is an indicator of changes in the pore structure. Older pavements often have hardly any interconnected pores and the sound absorption coefficients are correspondingly low.

#### Air resistivity

Based on ISO 9053−1 (2018)/ EN 29053, the airflow resistance of a surface layer is determined using a non-destructive measurement in order to obtain information on the ventilation in the tire/road contact area. These properties are of great importance with regard to the generation of noise when rolling over a surface layer.

To carry out the measurement, a rectangular chamber with an elastic sealing ring is pressed onto the pavement surface by means of a load. This is intended to create a situation similar to that which occurs when a tire rolls over the surface. A constant flow *q* through a defined area *A* is generated with a pressurized air regulator. The pressure difference Δ*p* generated by the flow through the surface is measured. The air flow resistance of a surface layer is defined as the ratio of the overpressure in the chamber Δ*p* to the flow rate *q*. The specific flow resistance *R*_s_ is defined as the ratio of the overpressure to the flow velocity *u* = *q*/*A* (*A* denotes the test area). This definition is explained in the following equation.

7 $$R_s=A\frac{\Delta p}q\;(\text{Pa}.\text{s}.\text{m}^{-1})$$

#### Acoustic simulation of test slabs using SPERoN

The measurement methods described above are also used for acoustic simulations (Kuijpers et al. 2007). With SPERoN, predictions can be made about the force distribution in the tire-road contact and predictions of tire vibrations, airflow noise in the tire-road contact, friction effects, tire noise, and aerodynamic vehicle noise. This makes it possible to compare the manufactured panels on the one hand with each other and on the other hand also in comparison with measurements in the field.

## Experimental results

Using a multi-scale experimental method, the mixtures were investigated from the mm-scale using the dynamic shear rheometer to the cm-scale using various laboratory tests to m-scale using the MMLS3 device.

### Mastic (mm-scale)

Various mastic combinations were investigated as outlined in the previous section. Unaged and aged mastic was investigated with all three fillers and two types of bitumen and an additive.

**Fig. 3:**
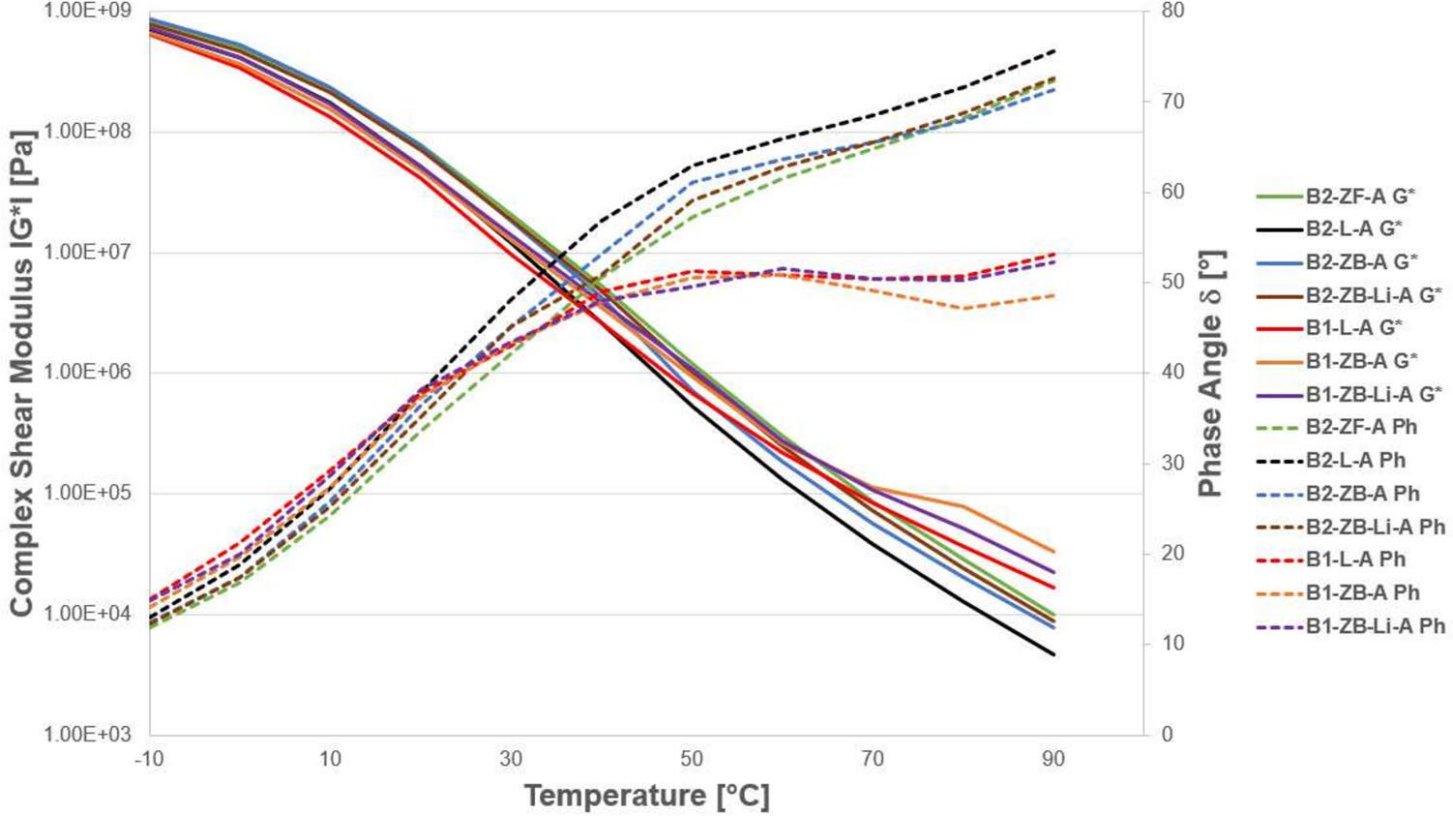
Effect of the addition of different fillers on aged (A) mastic and comparison with bitumen PmB 45/80–65 (B2) and HM (B1). For the abbreviations, see Table 2. The phase angle of the samples is the dotted line, the color is the same as the complex shear modulus, which is the solid line

A sample of the results shown in Fig. 3 indicates that Zeobit (ZB), Limestone (L), and Zeofill (ZF) have similar results on both types of bitumen at − 10 °C; however, at 30 °C, the influence of filler becomes apparent with a higher stiffening effect for Zeobit; this influence is larger as the temperature increases. The influence of filler at cold temperatures is minimal. Furthermore, the phase angle of the high modulus binder (HM) with Zeobit and lime remains low indicating a more elastic response at higher temperatures.

The optimal curve for the shear modulus would be as low as possible at − 10 °C and high at high temperatures. As Fig. 3 is in logarithmic scale, the real differences between the curves are hard to judge. Therefore, in Table 3 is a comparison of all the aged results to the aged B2-L (PmB-45/80–65 with Limestone). The table shows the ratio of the measured value of a mixture to the value of the mixture B2-L. For example, the value 2.1 for B2-ZF-A/90 °C means that the B2-ZF-A mixture has a value 2.1 times higher than the B2-L mixture.

**Table 3:** Comparison of all the aged mixtures to the PmB-45/80–65 Limestone (B2-L-A), see designations in Table 2, *A* aged (for abbreviations, see Table 2)

	B2-ZF-A	B2-ZB-A	B2-ZB-Li-A	B1-L-A	B1-ZB-A	B1-ZB-Li-A
[°C]						
-10	1.2	1.2	1.1	0.9	0.9	1.0
30	1.7	1.5	1.6	0.8	1.0	1.2
60	2.3	1.4	1.9	1.7	2.0	2.1
90	2.1	1.7	1.9	3.6	7.0	4.8

**Fig. 4:**
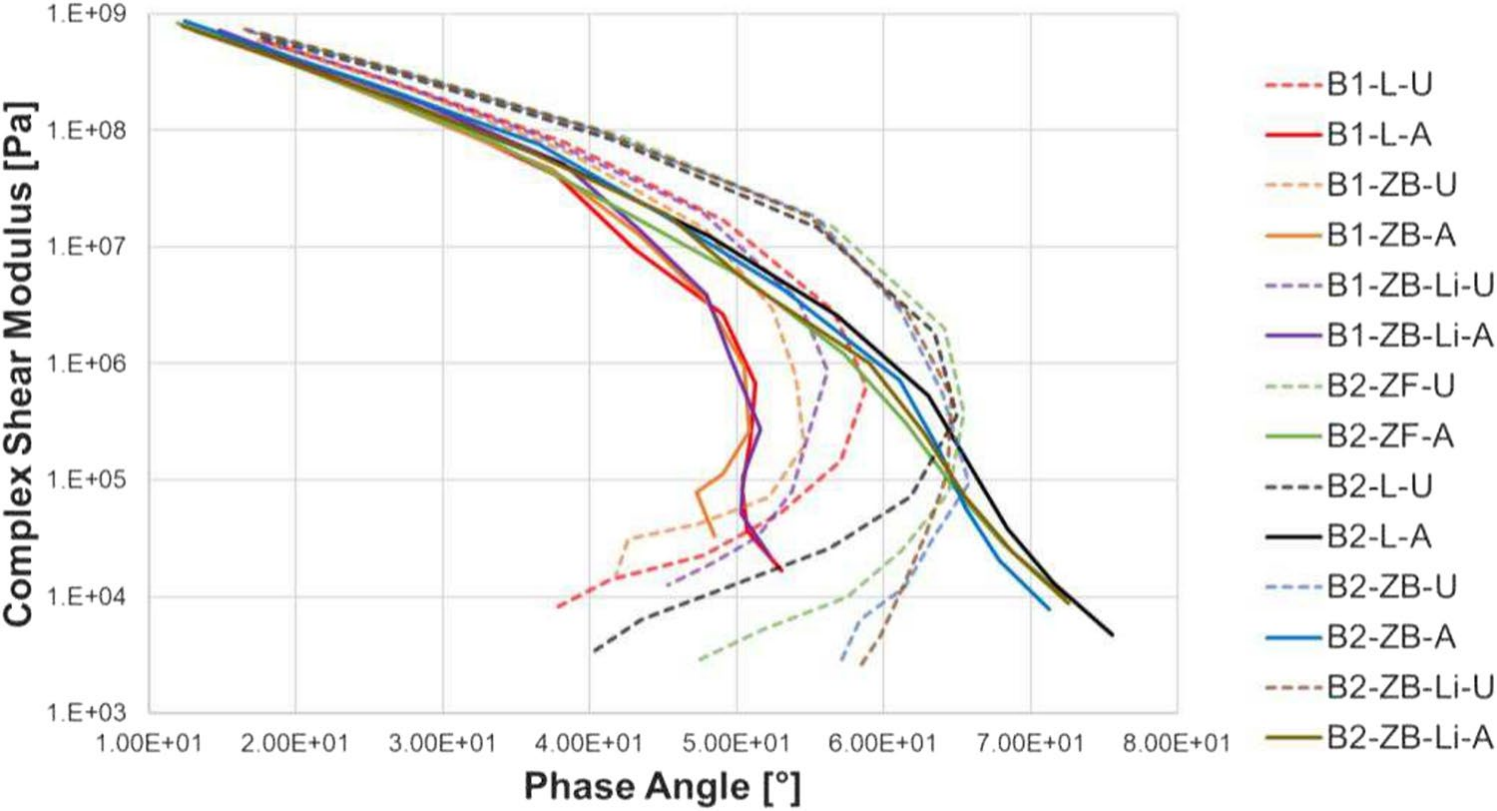
Black diagram showing the effect of the different fillers on unaged (dotted line) and aged (solid line) mastic and comparison with bitumen PmB 45/80–65 and HM

Figure 4 shows the Black Diagram with the complex shear modulus versus the phase angle. This type of diagram allows to compare the materials without the effect of temperature and frequency. The general pattern observed is a reverse C shape which is what is normally seen for an SBS polymer-modified binder. This was true for all materials except aged PmB with the three fillers. The HM binder was able to retain its reverse C shape even after aging. In general, aging resulted in shifting of the data towards the left, i.e., lower phase angles indicating a more elastic response after aging, with the curves of aged HM with Zeobit and lime shifting the most indicating the lowest phase angle.

The main findings from the mastic experiments can be summarized as follows:

1. Addition of filler increases the complex modulus in the whole temperature range and the phase angle primarily above 30 °C.
2. The highly modified binder had a higher modulus and lower phase angle in comparison to the conventional PmB primarily above 30 °C.
3. Aging increases the complex modulus in the whole temperature range and decreases the phase angle below 50 °C and above which an increase is seen.
4. In unaged state and at cold temperatures, the influence of the three fillers on the conventional binder PmB 45/80–65 (B2) is similar. This is evident when results of Zeobit, Limestone, and Zeofill at − 10 °C are compared.
5. In unaged state and intermediate temperatures, Zeobit was the stiffest and the highest phase angle above 30 °C. At 90 °C, sandstone filler had the highest modulus.
6. The influence of the different fillers at cold temperatures was minimal and its influence was highest at intermediate temperatures. This is understandable as at cold temperatures the composite material is elastic whereas at higher temperatures, the binder becomes soft and viscoelastic whereas the filler remains elastic and its influence becomes more apparent.
7. The aged highly modified bitumen with limestone filler and Zeobit and Zeobit with lime modifier show the best results and similar results in the mastic scale.
8. The additive lime did not influence the ageing process significantly. However, the ageing procedure used is only adding oxygen to the mix. Water and UV influence cannot be simulated with the PAV procedure.

### Mixture (cm-scale)

#### Indirect tensile strength and water sensitivity of benchmark mixtures

**Fig. 5:**
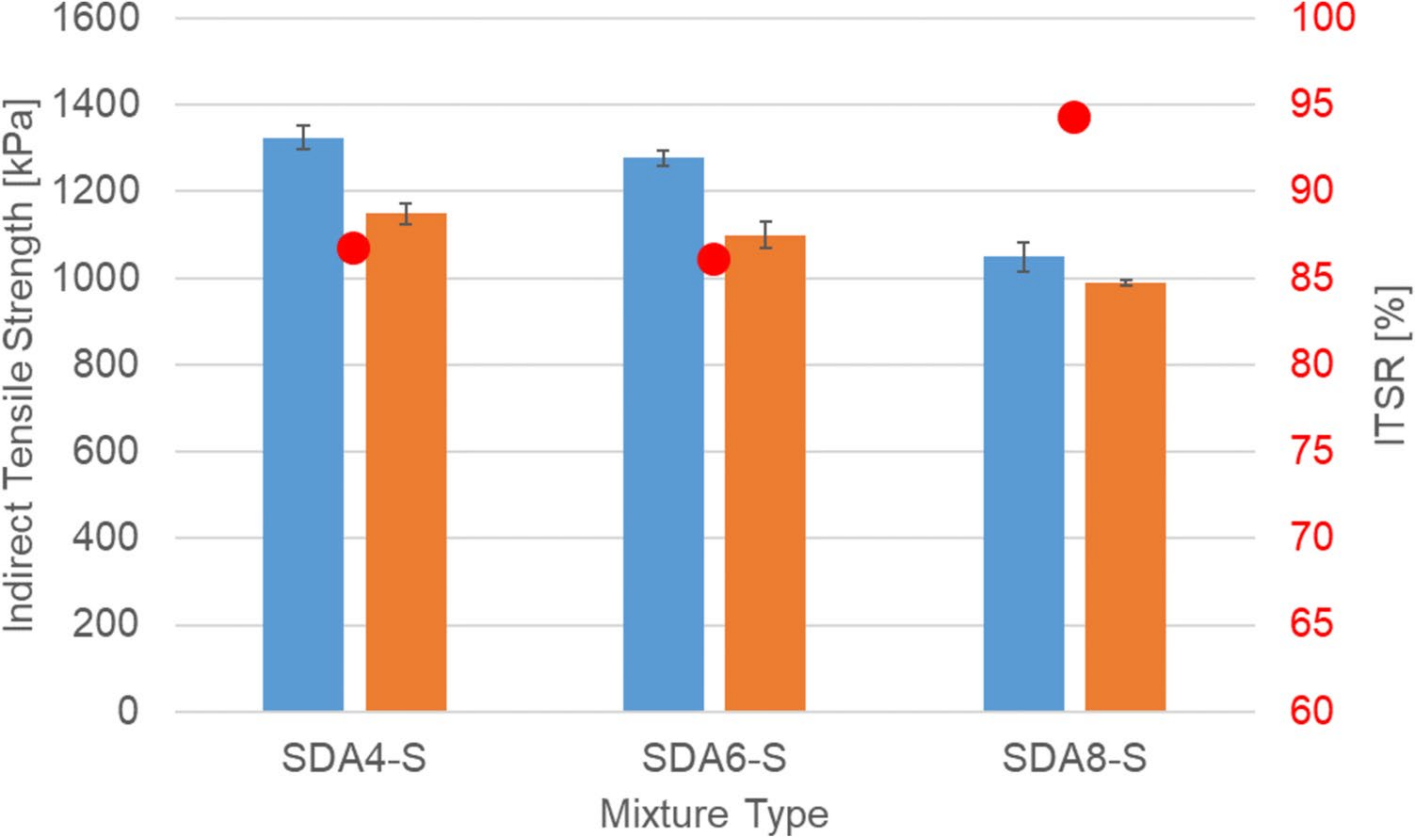
Dry and wet strength and water sensitivity (red dots) of benchmark SDA 4, SDA 6, SDA 8

Figure 5 shows the indirect tensile strength in dry state and after being water conditioned referred to as wet state. Three replicates were tested for each mixture and condition. As shown in the figure and as expected, the dry strength is higher than the wet strength and the ranking of the mixtures is SDA 4 > SDA 6 > SDA 8 in all cases. It is to be noted that the repeatability of results should be 15% according to the standard and therefore the difference between SDA 4-S and SDA 6-S is negligible. It should be noted that SDA 8 has performed better in situ. The difference here can be attributed to the fact that there is a lateral support exerted by the material in situ whereas in lab experiments this does not exist. Additionally, the larger void size also reduces the mechanical strength of the sample.

The water sensitivity values (ITSR) of the benchmark mixtures is shown in. All benchmark mixture pass the Swiss required ITSR of 70% minimum.

**Fig. 6:**
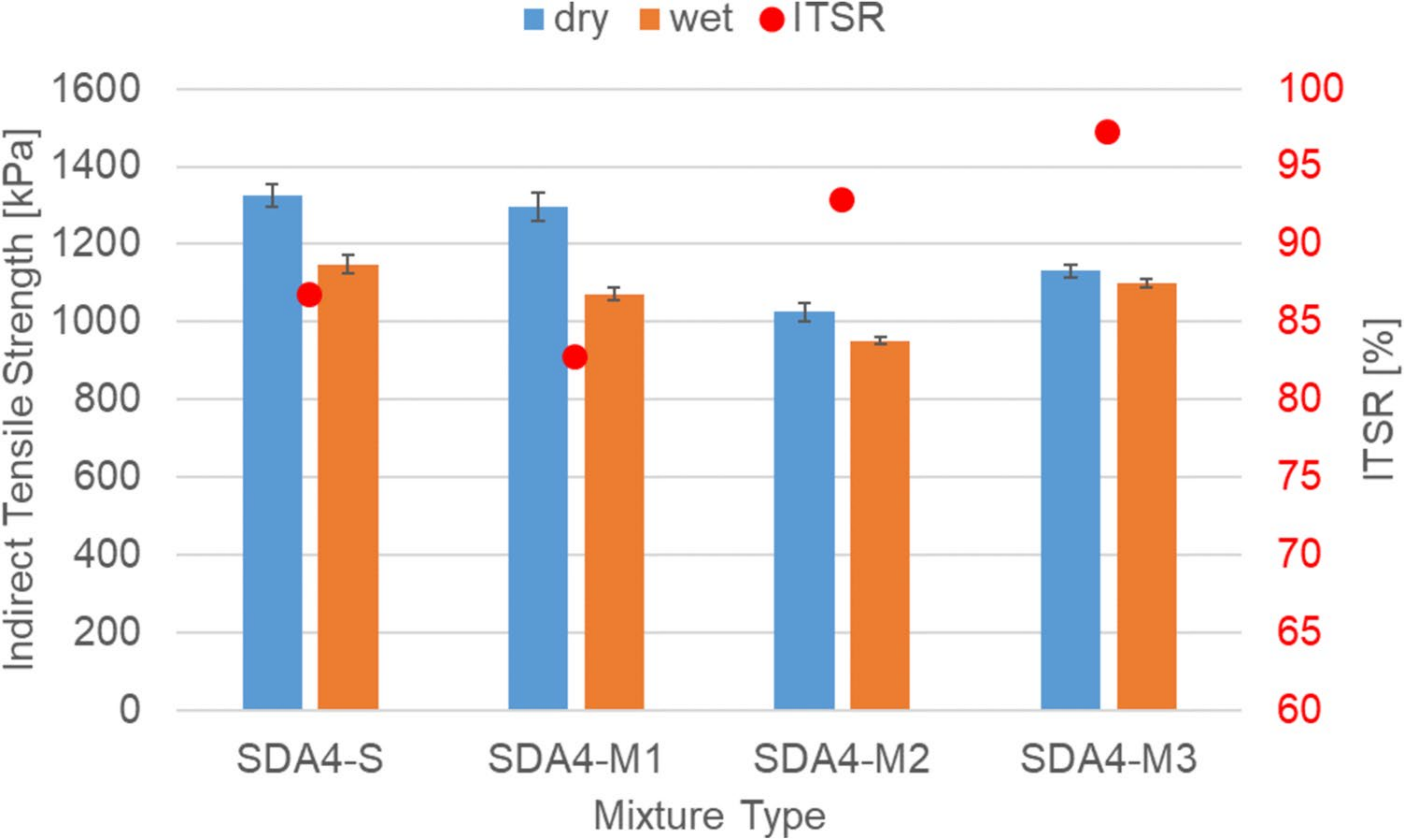
Dry and wet strength and water sensitivity (red dots) of SDA 4-S and three modified mixtures SDA 4-M1, SDA 4-M2, and SDA 4-M3

Figure 6 shows the water sensitivity of the SDA 4-S benchmark mixture in comparison to the three modified ones SDA 4-M1, SDA 4-M2, and SDA 4-M3. The figure shows that all mixtures satisfy the Swiss requirement of ITSR > 70%. Mixture SDA 4-M1 shows very similar dry and wet strength in comparison to the SDA-S mixture but slightly lower water sensitivity values. It can be seen from Fig. 6 that the binder and filler have reduced the wet strength and water sensitivity values of SDA 4. SDA 4-M3 has somewhat lower strength in comparison to SDA 4-S but very little difference between wet and dry strength leading to very high ITSR of 97%.

#### French rutting test

**Fig. 7:**
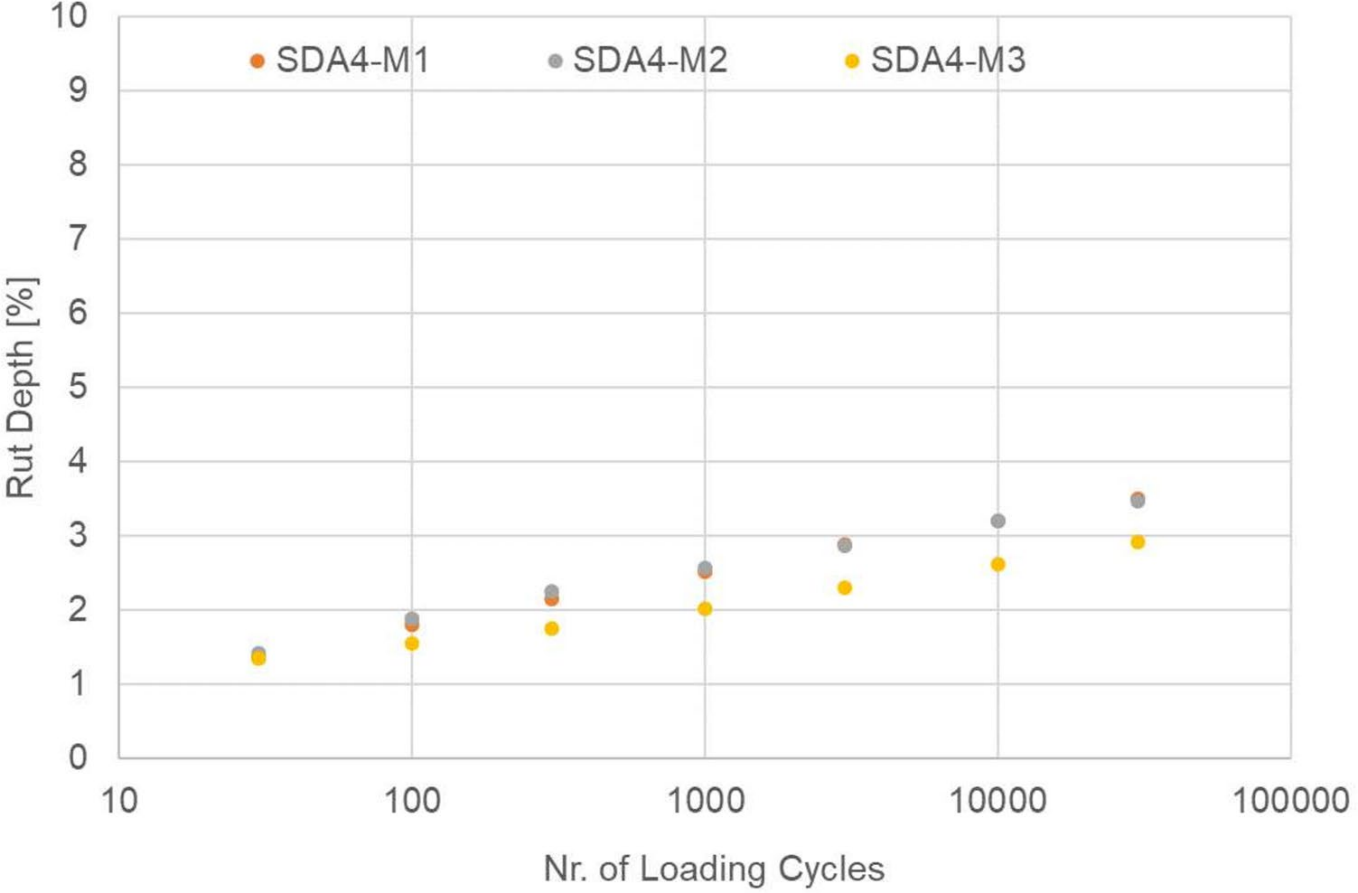
French rutting test results for SDA 4-M1, SDA 4-M2, and SDA 4-M3

The French rutting test was performed on the modified mixtures in order to guarantee that these mixtures pass the rutting test. The requirement of the SN indicates that the value needs to be reported however, no threshold is defined. The lowest threshold for other types of mixtures is 7%. Figure 7 reports the results of the three mixtures after 30,000 cycles. As shown, the amount of rut is minimal at 4% at 60 °C. The ranking of the modified mixtures after 30,000 cycles is SDA 4-M3 > SDA 4-M2≈SDA 4-M1.

#### Cyclic compression test

**Fig. 8:**
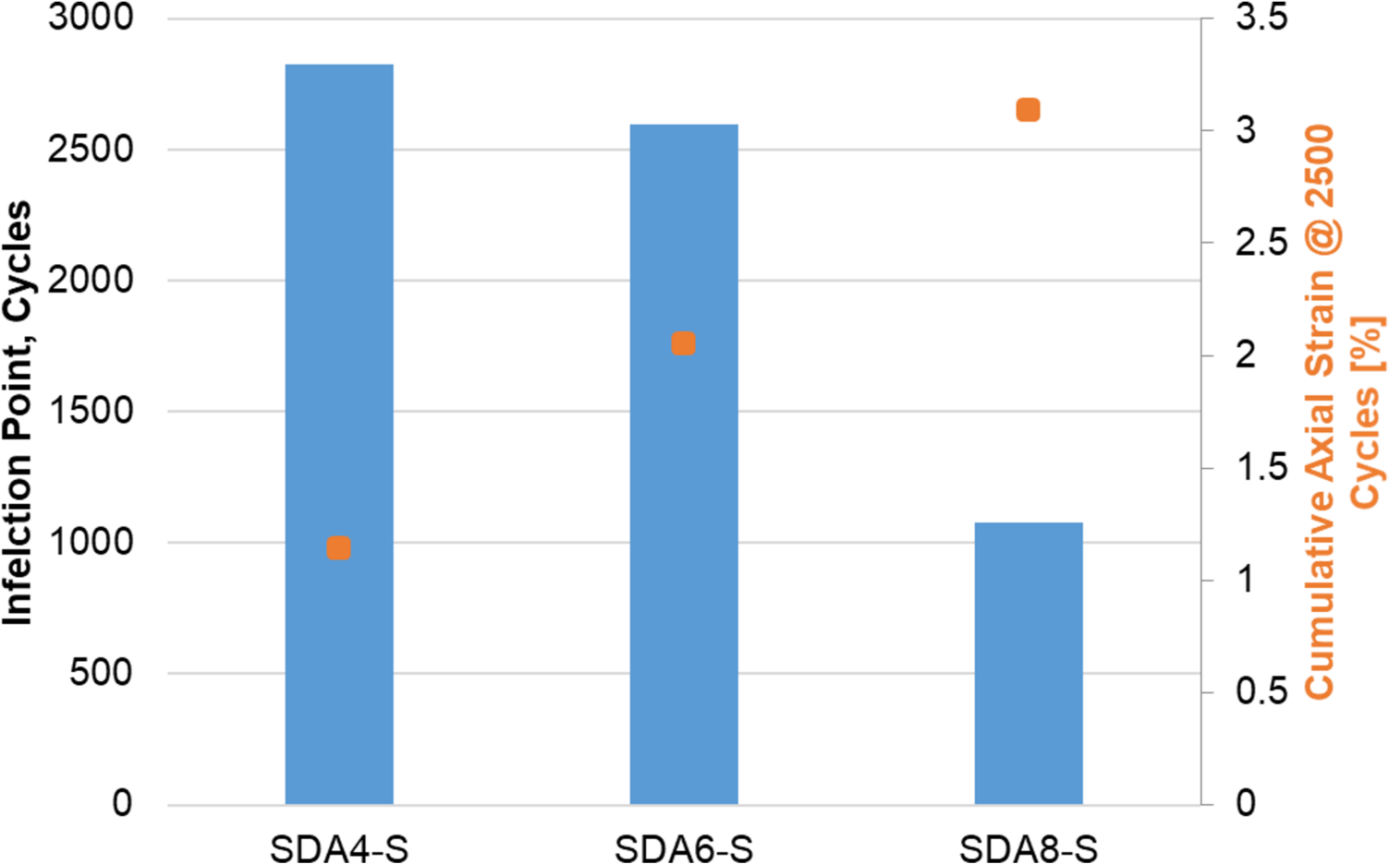
Inflection point and cumulative axial strain at 2500 cycles for SDA 4-S, SDA 6-S, and SDA 8-S

The mixture creep was tested using the cyclic compression test (CCT) at 50 °C. The inflection point and cumulative axial strain at 2500 cycles for the three standard mixtures are shown in Fig. 8. It can be seen that SDA 8 reached the inflection point after much fewer cycles in comparison to SDA 6 and SDA 4. The cumulative axial strain was also much higher for SDA 8. The ranking for the CCT is as follows: SDA 4-S > SDA 6-S > SDA 8-S. As discussed above, the higher void content and lack of lateral support have a strong effect on the results of this test.

**Fig. 9:**
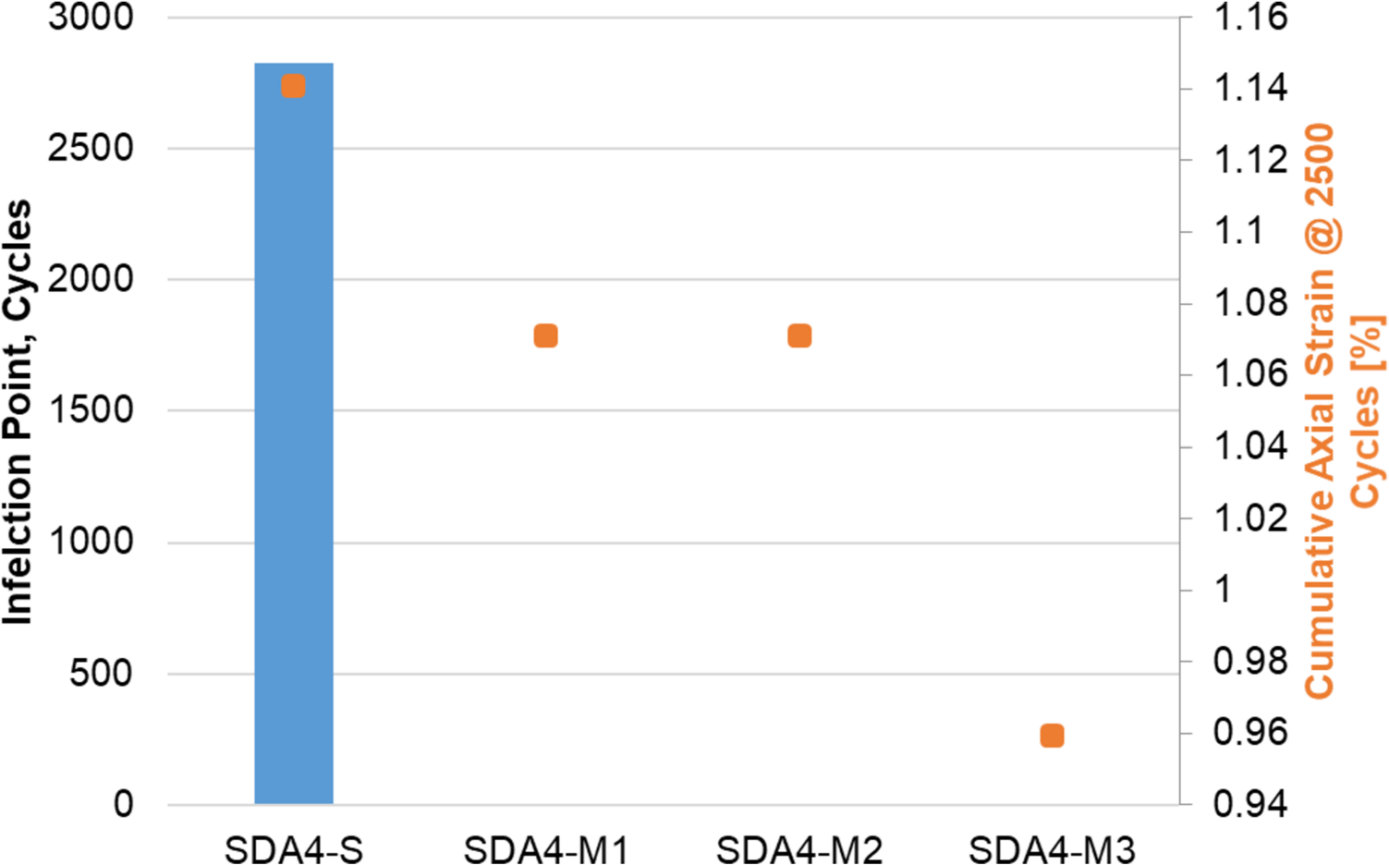
Inflection point and cumulative axial strain at 2500 cycles for SDA 4-S, SDA 4-M1, SDA 4-M2, and SDA 4-M3

The results of the CCT for the modified mixtures in comparison to the SDA 4-S is shown in Fig. 9. It can be seen that at higher temperatures the modified mixtures all had no inflection point and much lower cumulative axial strain in comparison to the standard mixture SDA 4-S. M1 and M2 had similar performance in this test; however, the performance of M3 was significantly better in terms of cumulative axial strain. The results of this test corroborate the mastic results that the modifications improve the creep performance of the mixtures.

#### Semi-circular bend test

**Fig. 10:**
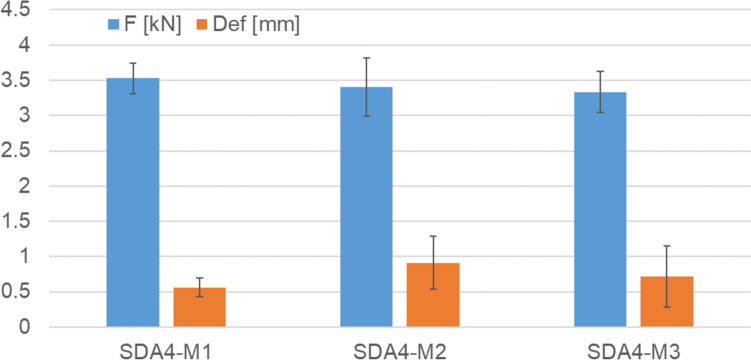
Maximum force (F) and maximum deformation (Def) for the SCB test at 0 °C. The error bars indicate the standard deviation of the results for four samples

In order to test the resistance of the mixtures to low temperature cracking, four samples were tested at 0 °C for each material type. Figure 10 shows that although the maximum force to failure was similar for the three mixtures, the deformation for M2 and M3 was larger indicating a slightly more ductile behavior that is positive in preventing catastrophic failure during cracking. Although in Switzerland no thresholds exist for the SCB test, the order of magnitude of the maximum force to failure of ca 3.5 kN and deformation of ca 0.4 mm is within the magnitudes that were seen in tests on SDA mixtures.

#### Stiffness modulus

**Fig. 11:**
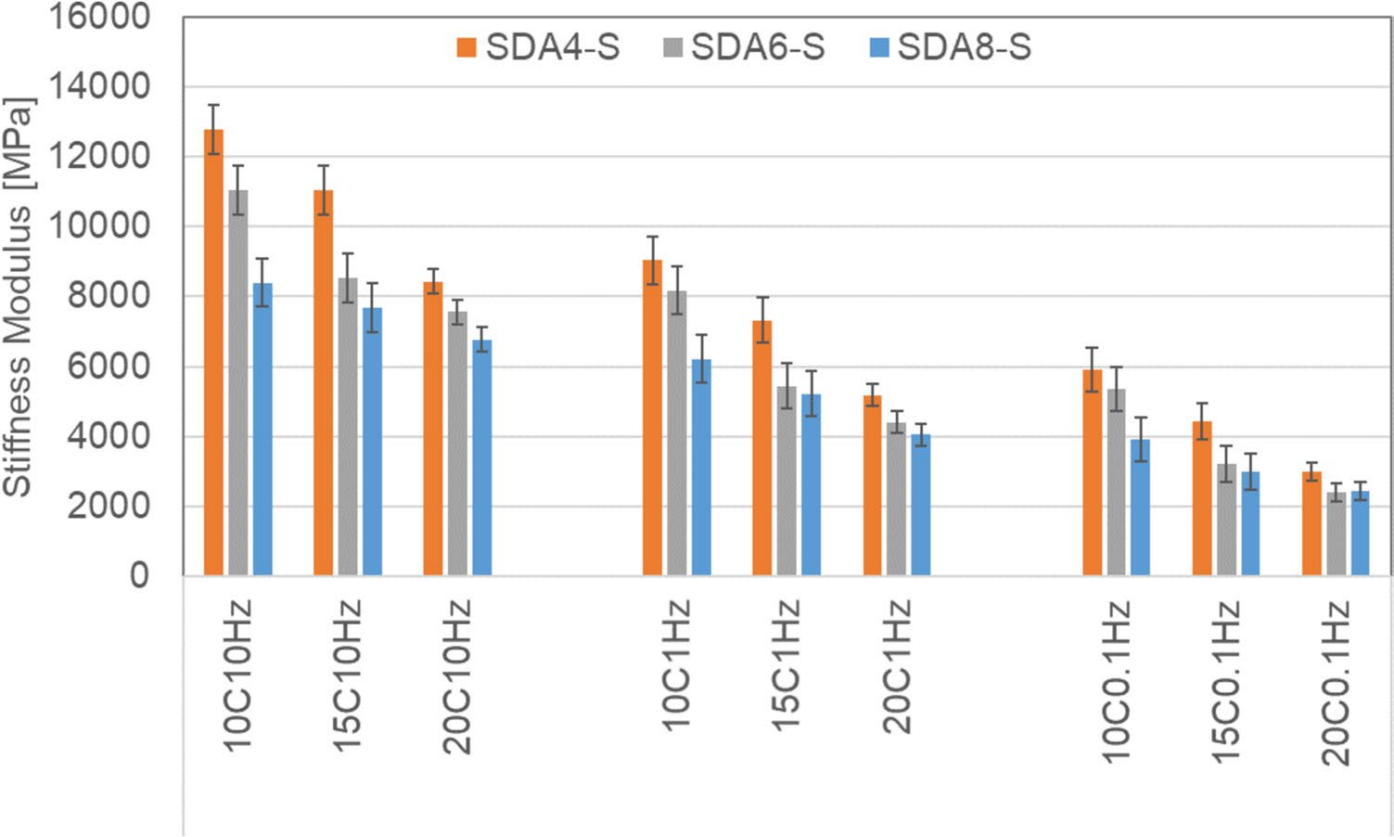
Stiffness modulus of benchmark SDA 4-S, SD6-S, SDA 8-S

The stiffness modulus of the benchmark mixtures measured using the cyclic indirect tensile test on cylindrical samples under sinusoidal load (CIT-CY) is shown in Fig. 11. Four replicates were tested and three temperatures (10, 15, 20 °C) and three frequencies (0.1, 1, 10 Hz) were used for these tests. The ranking of the tested benchmark mixtures were as follows: SDA 4 > SDA 6 > SDA 8 for all temperatures and frequencies tested. Here by ranking the higher value is meant as higher modulus is not necessarily better when we consider performance as this would depend on the context.

**Fig. 12:**
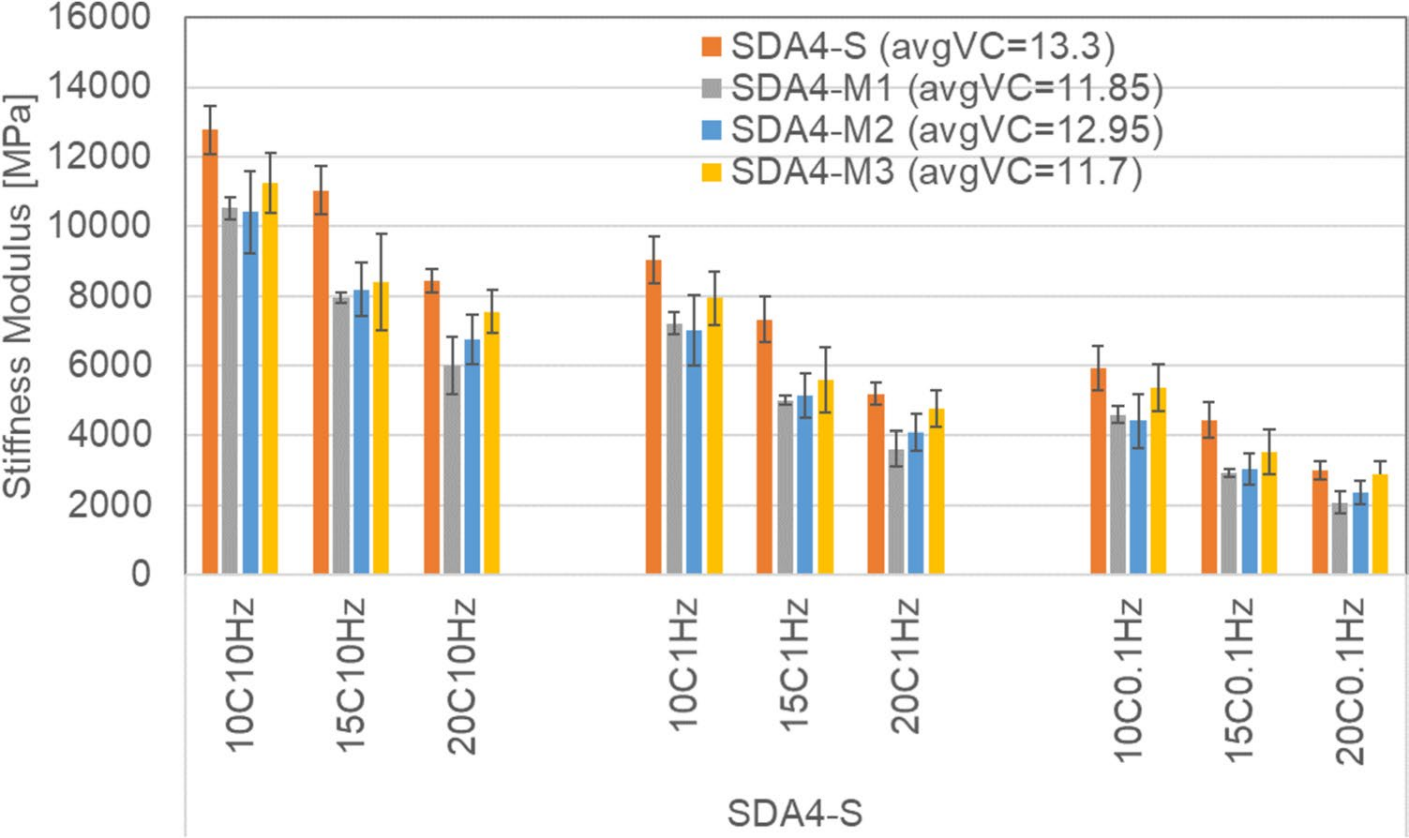
Stiffness modulus of benchmark SDA 4-S, and modified SDA 4-M1, SDA 4-M2, and SDA 4-M3

Figure 12 shows the stiffness modulus of SDA 4 benchmark mixture in comparison to the modified ones SDA-M1, SDA 4-M2, and SDA 4-M3. The figure also shows the average air void content for the four samples used for each mixture. The results show that even though the benchmark mixture S has the highest voids content, for all temperatures and frequencies, the benchmark mixtures showed a higher modulus with this difference being smaller at lower frequency of 0.1 Hz and higher temperature 20 °C. Amongst the modified mixtures, M3 had on the average a higher modulus. As seen in the mastic results, the highly modified binder used for M2 and M3 shows a difference with the other binders at higher temperatures. At the mixture scale, it can be seen that the modulus of the M3 mixture in comparison to M1 and M2 is the highest at 20 °C. As M2 and M3 have the same highly modified binder, this difference can be attributed to the fillers used in M3, i.e., a combination of Zeobit and lime.

#### Fatigue

**Fig. 13:**
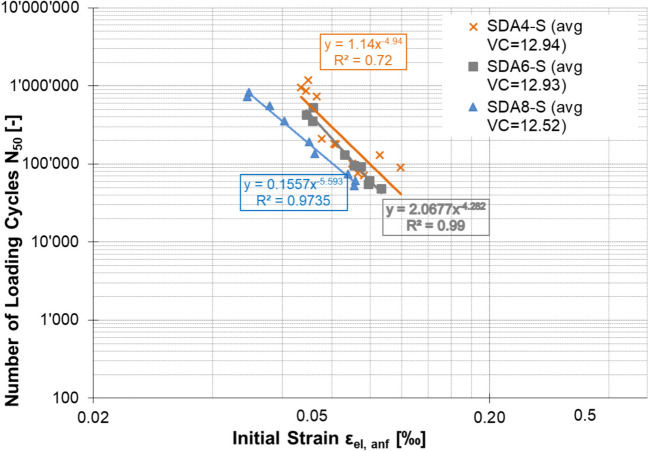
Fatigue curve of benchmark SDA 4, SDA 6, SDA 8

**Fig. 14:**
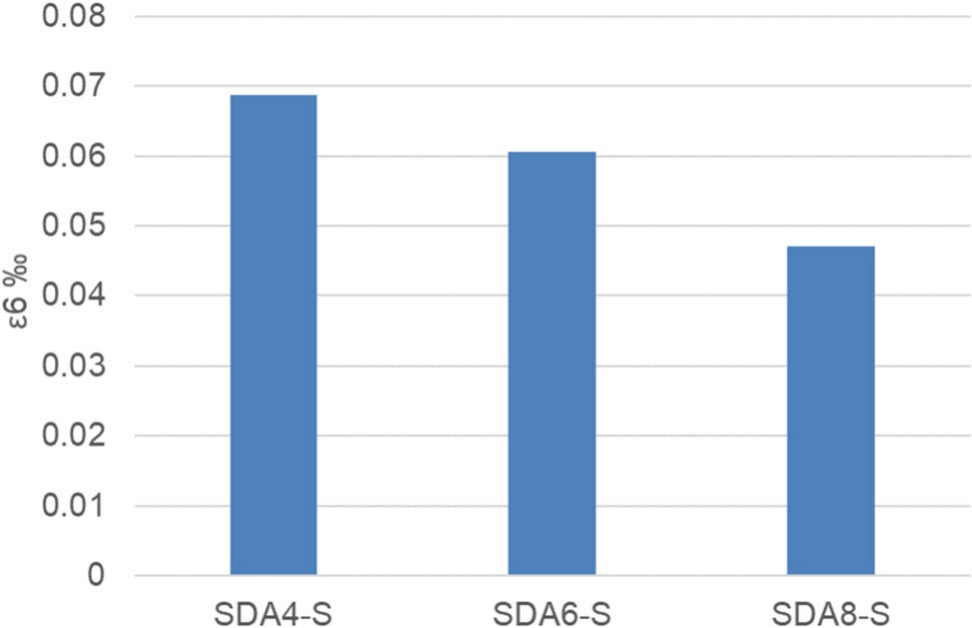
Fatigue performance of benchmark SDA 4, SDA 6, SDA 8

The results of the fatigue experiments on cylindrical samples of the benchmark mixtures under sinusoidal load at 10 °C and 10 Hz are shown in Figs. 13 and 14. As seen in the fatigue curve and ε_6_ parameter, the fatigue performance of the benchmark mixtures reflects previous results with a similar ranking: SDA 4 > SDA 6 > SDA 8.

**Fig. 15:**
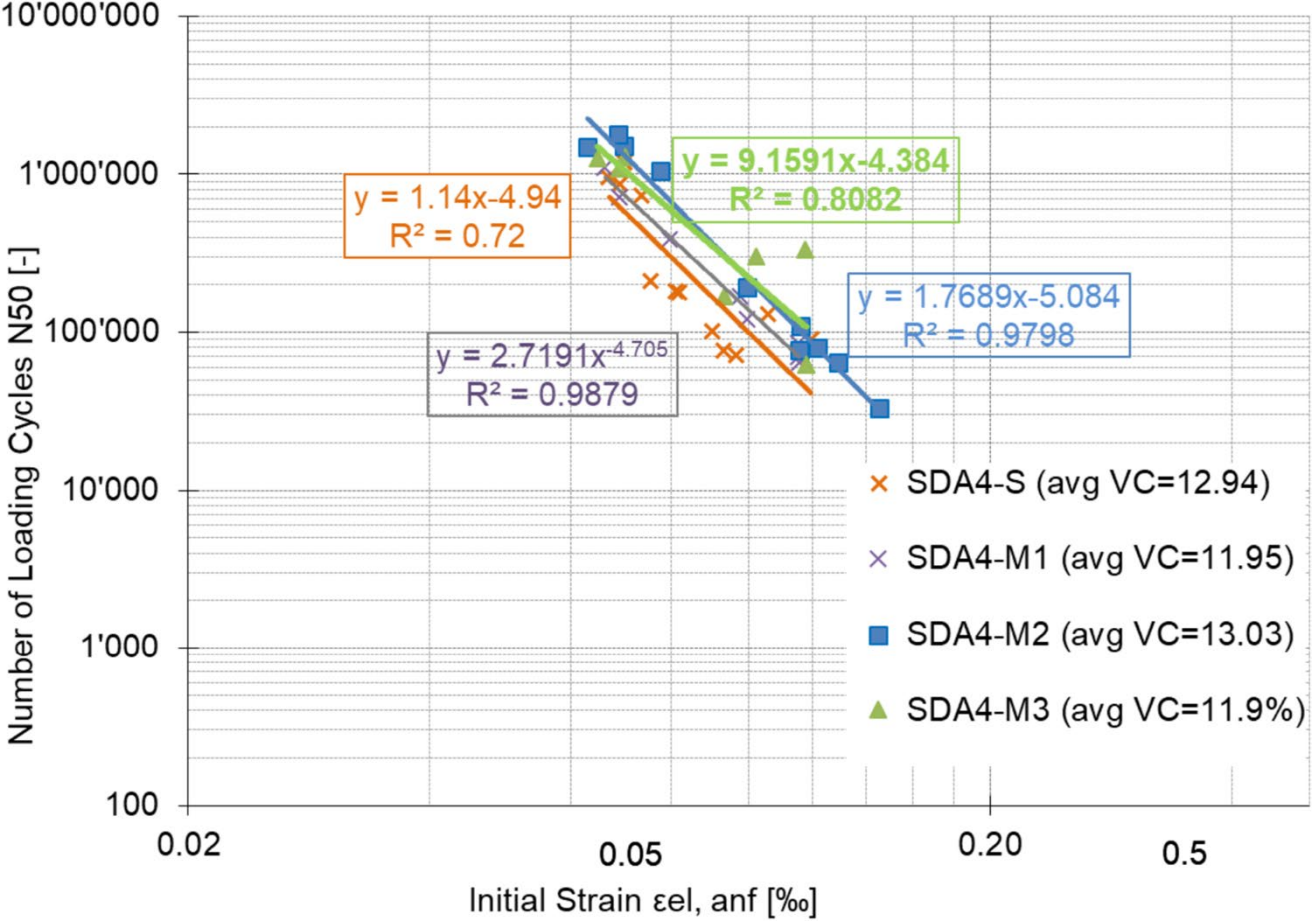
Fatigue performance of standard and modified mixtures SDA 4-S, SDA 4-M1, SDA 4-M2, SDA 4-M3

**Fig. 16:**
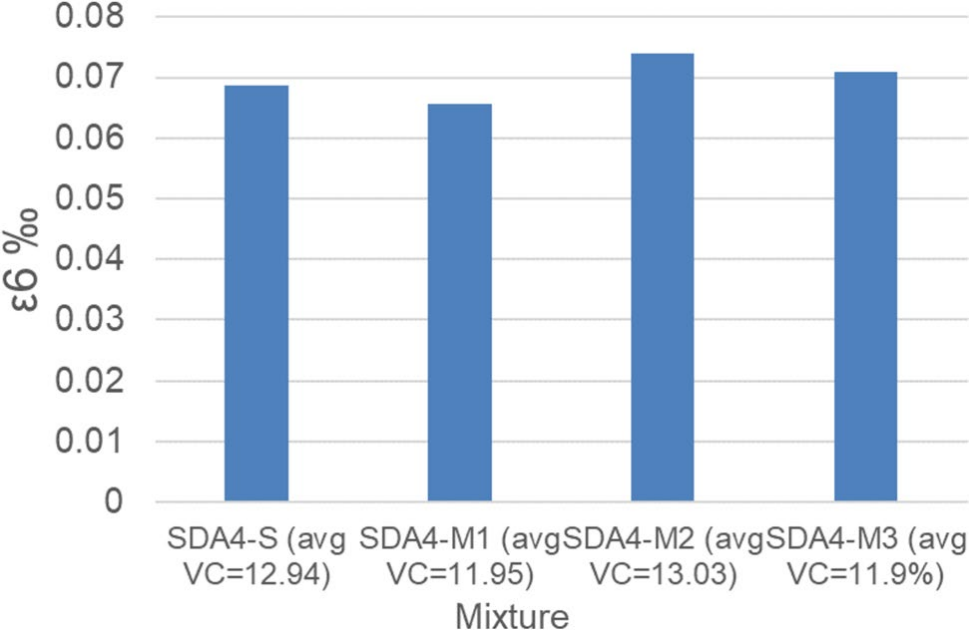
Fatigue performance in terms of ε_6_ of standard and modified mixtures SDA 4-S, SDA 4-M1, SDA 4-M2, SDA 4-M3

The fatigue performance of the modified mixtures and comparison to SDA 4-S are shown in Figs. 15 and 16. The figures show that the fatigue performance of the modified mixtures is better than the standard mixtures. The ε6 parameter decreased for M1 (− 4.6%) and increased for M2 (7.4%) and M3 (3.2%) in comparison to SDA 4-S.

#### Particle loss: MDI

**Fig. 17:**
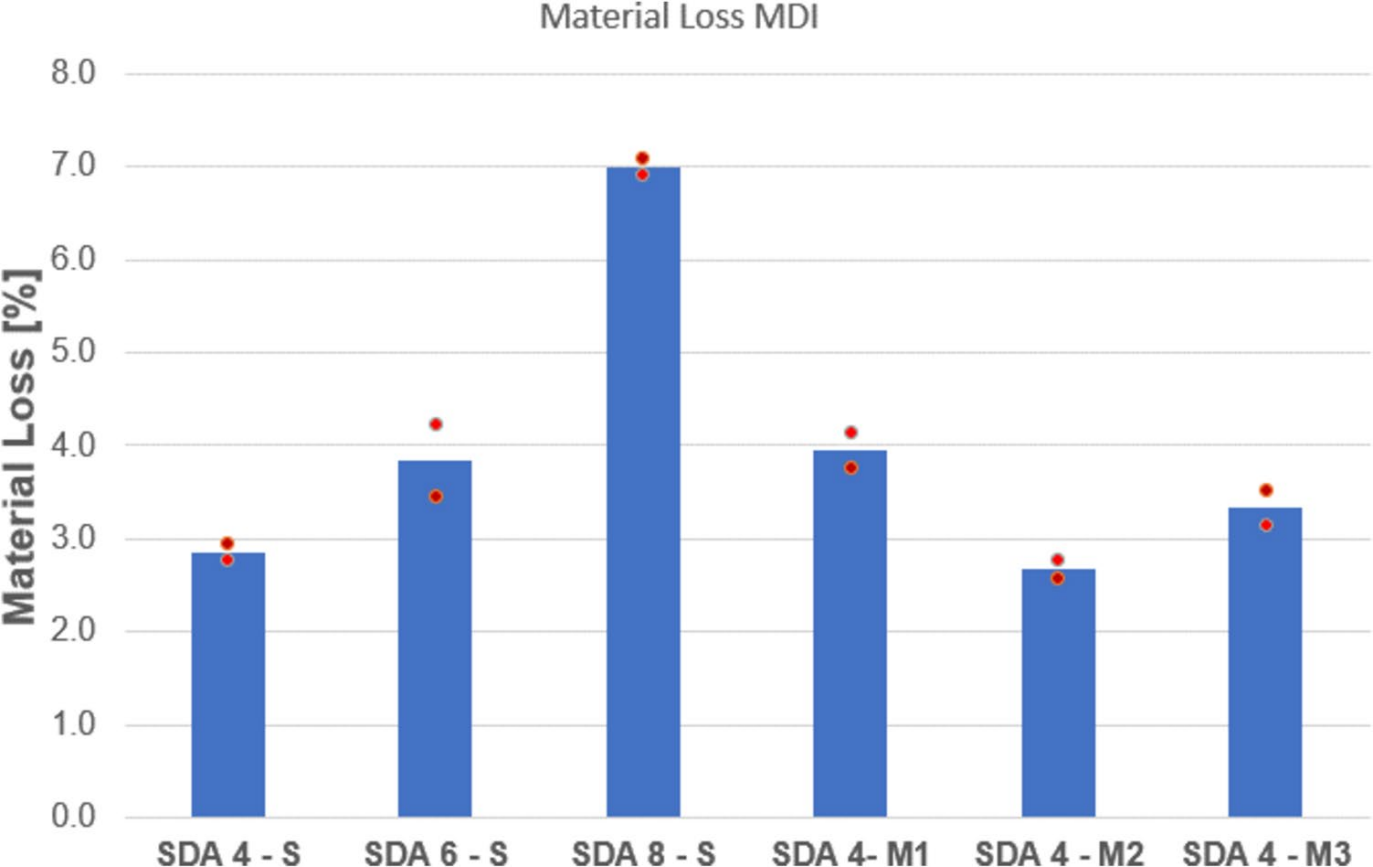
Particle loss according to the MDI test

The test was carried out on a total of 6 mixtures. As shown in the results in Fig. 17, the reference mixtures’ particle loss ranking was as follows: SDA 4 < SDA 6 < SDA 8. This is partly due to the fact that the larger particles also have higher weights and result in a higher mass value. All mixtures with the exception of SDA 8 had particle loss values under 4% which is low in comparison to allowable values for the European standard Cantabro test which is normally 10%.

The following conclusions can be drawn from the MDI test:

- The differences between the materials are significantly greater than the dispersion of the two individual values. The differences are therefore mostly relevant.
- The reference mixtures clearly show that the loss in mass increases with increasing maximum grain size. However, it should be noted that in the laboratory test, the mass of the test specimens is the same for all mixtures. This means that fine-grained SDA 4 mixtures tend to be favored, as the loss of a single grain is significantly smaller than with an SDA 8.
- Based on experience with this test, differences in the order of 0.5 mass % are insignificant. This means that the SDA 4-M2 and SDA 4-M3 mixtures should be evaluated similarly to the reference mixture. SDA 4-M1 performs slightly worse than the reference mixture and the mixtures SDA 4-M2 and SDA 4-M3 in terms of grain loss.

#### MMLS3 rutting performance (m-scale)

The mixtures that were tested at cm-scale were further tested in a larger scale using slabs. In this case, the mechanical durability is combined with acoustic performance. To this end, mechanical durability was tested by recording the developing rut depth with the number of loading cycles using the MMLS3 traffic simulator. On the other hand, air resistivity, sound absorption, and surface texture were measured to determine the acoustic behavior in the wheel path and outside the wheel path.

**Fig. 18:**
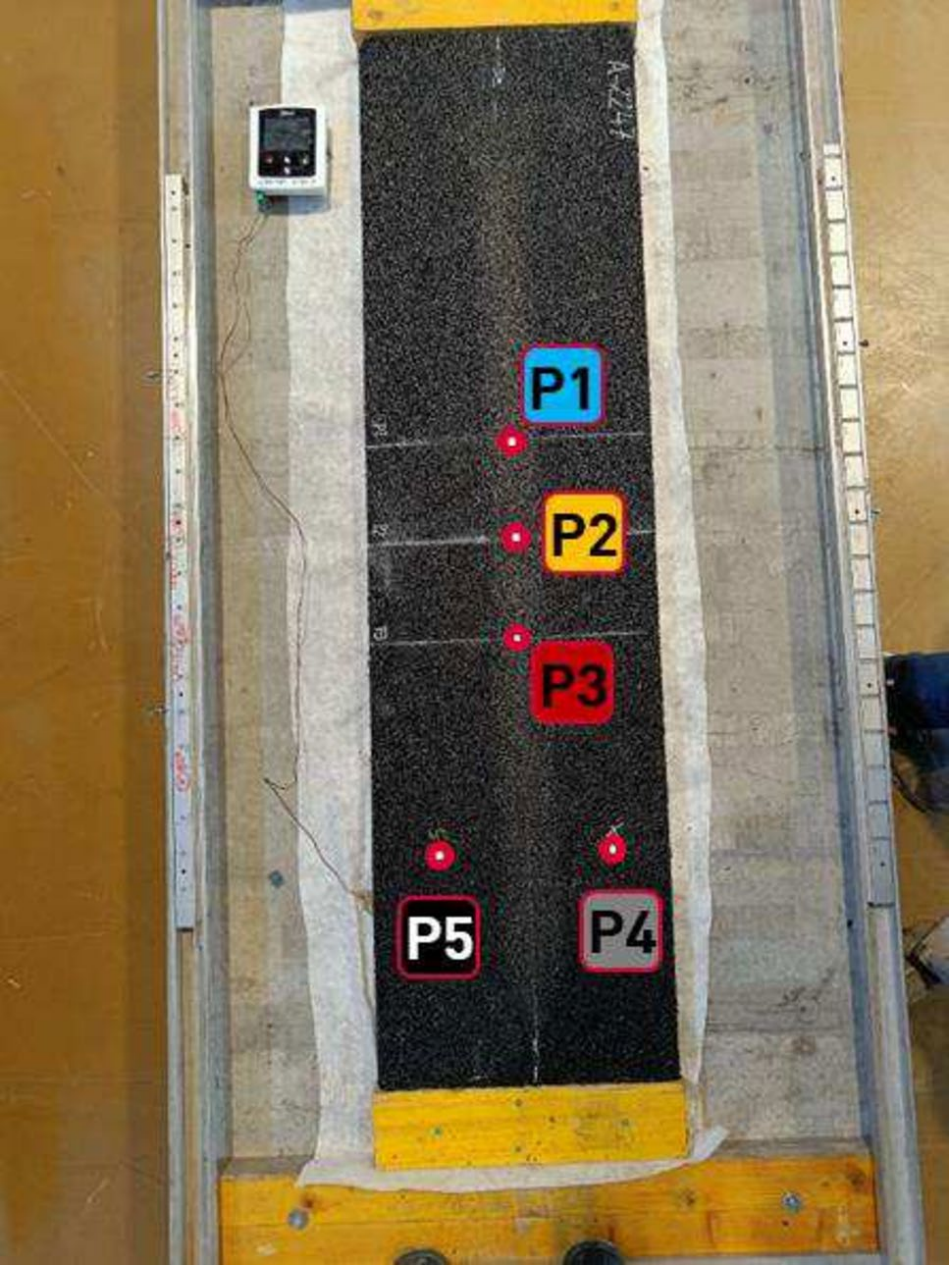
Measurement point setup of the MLSS3 slabs

Many of the tests performed on the test slabs are point measurements measuring a characteristic on an isolated spot (Skov et al. 2015). In order to have multiple measurements per plate and to isolate possible differences between the measurements, multiple measurement point were defined (see Fig. 18). There were three points in the wheel path (P1, P2, and P3) and two points outside the wheel path (P4 and P5) which could be used as a reference. For the destructive tests, which are performed after the MLSS3, the points P1 and P4 are used.

For each mixture, two slabs were tested. In one of the slabs, only rutting measurements with the profilometer were carried out at different loading intervals up to 64,000 MMLS3 load applications. It was seen, however, that most of the rutting happened in the initial loading phase and after that rut depth was not increasing significantly, so for the rest of the materials the loading was limited to 50,000 load applications. In the other material slabs, the rutting was measured simultaneously with texture, air resistivity, and sound absorption after 0, 500, 1000, and 6000 MML3 load applications, where most of the changes in the material were taking place.

**Fig. 19:**
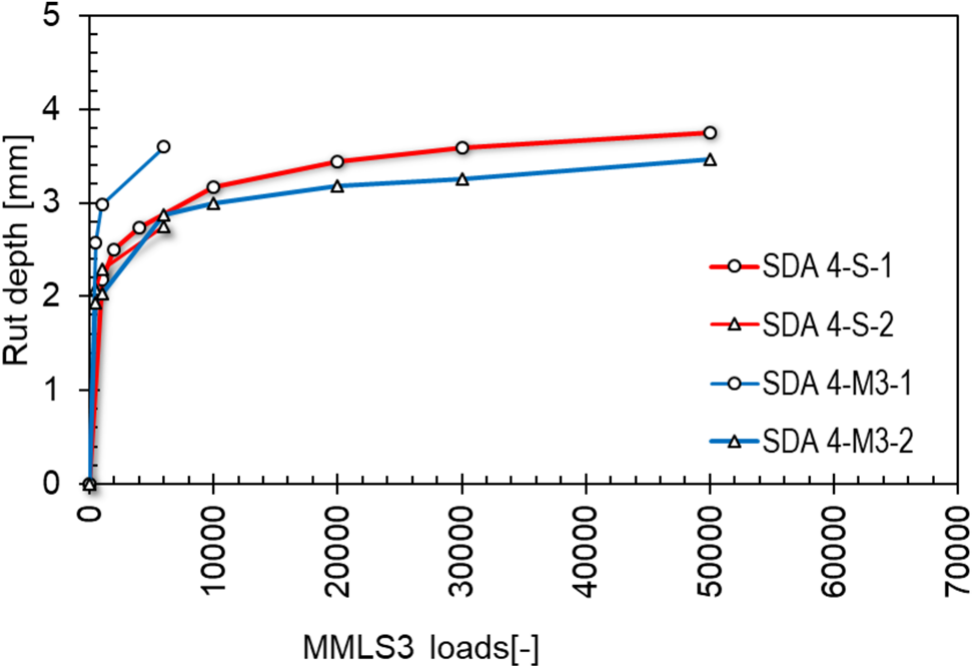
Development of rutting of two slabs of benchmark SDA 4-S and modified SDA 4-M3 using MMLS3

**Fig. 20:**
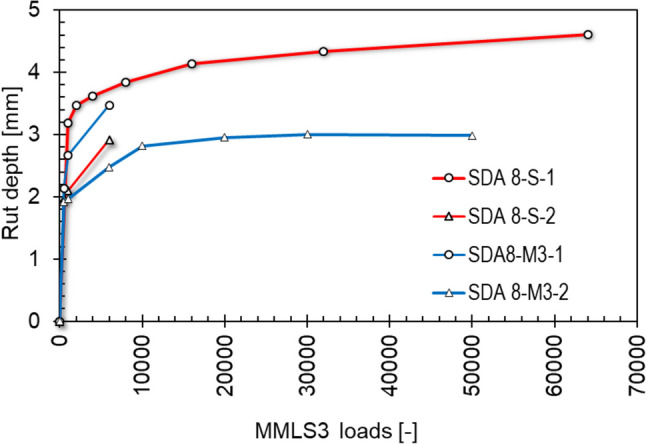
Development of rutting of two slabs of benchmark SDA 8-S and modified SDA 8-M3 using MMLS3

Figure 19 shows the development of the rut depth vs. the number of MMLS3 cycles for the SDA 4-S benchmark mixture and the modified mixture SDA 4-M3. Figure 20 shows the same results but for SDA 8-S benchmark mixture and the modified mixture SDA 8-M3. The figures show that the behavior of the two slabs with the same material is not always following the same path. The difference in the two slabs can be partially attributed to the change in loading temperature, as the shorter experiments (up to 6000 MMLS3 load repetitions) were interrupted regularly for up to an hour to make acoustic, air resistivity and texture measurements. To that end, the slabs were taken outside the temperature chamber, whereas for the longer experiments the slabs stayed in the chamber because profile measurements take only a few minutes and can be done with the MMLS3 in testing position. This means that the longer experiments are more reliable in terms of the effect of temperature in the rutting behavior. These show that both modified mixtures shown in blue have lower deformation compared to the benchmark standard mixtures (shown in red). Furthermore, the difference was much larger for SDA 8 compared to SDA 4. The large-scale experiments show that there is no adverse effect in using the modified mixtures to the rutting properties, and in fact, there is possibly some improvement.

##### MMLS3 acoustic tests (m-scale)

The MMLS3 test allowed to investigate several acoustically relevant parameters as listed in the sections as follows.

#### Surface texture

**Fig. 21:**
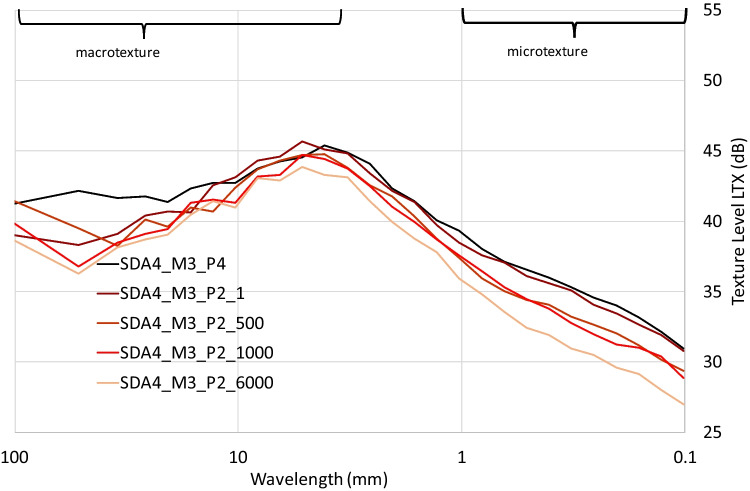
Surface texture level measurements for SDA 4 modified MMLS3 sample inside (P2) and outside (P4) of the wheel path

The surface texture data for the SDA 4 and SDA 8 MMLS3 slabs before loading show that the texture level of the SDA 8 samples are higher than that for the SDA 4. This is the case for the macrotexture where it would be expected given the higher aggregate size, but also in the microtexture. Looking at the effects of the MMLS3 wheel loading, the texture level for the SDA 4 and SDA 8 samples are shown in Figure 21, with the sample position and number of cycles indicated. The texture level in the wheelpath is reduced from the MMLS3 loading relative to the texture after compaction (P4 and P2_x cycles), which is indicative of the effect of the traffic on the surface texture of SDA, and has also been observed in field samples. The texture reduction is more significant for the SDA 4 sample, where the modified SDA 4 seems to have more resistance to reduction in the microtexture. This could indicate better performance in skid resistance, as higher microtexture is correlated with this parameter (Paje et al. 2008). However, we must keep in mind that this was only from comparing one slab per mixture and that the effect of the texture when including the effects of weathering in situ are more complex (Kuijpers et al. 2007). Overall, the modification of the SDA mastic did not have a significant effect on the texture properties before and after wheel tracking.

#### X-ray tomography

**Fig. 22:**
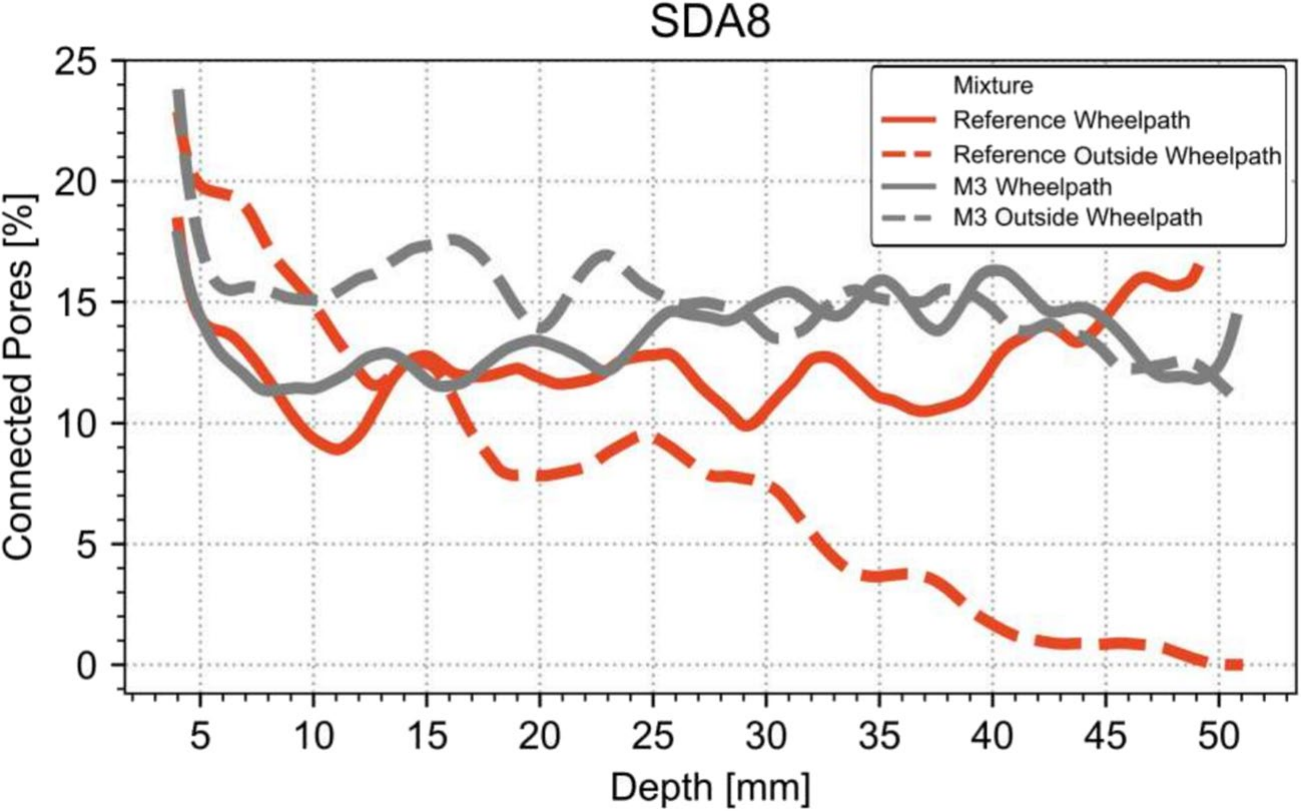
Connected pore network for the SDA 8-Slabs. Red: reference slab; gray: optimized mixture (M3). Solid lines: MP2: Wheelpath after MMLS (6000 cycles) MP4: Outside wheelpath (dotted lines)

Figure 22 shows the profile of the void content connected to the surface over the depth of the drill core evaluated with X-ray tomography. The reference slab (not shown) and the optimized slab can have very different behavior in terms of connected void contents. While connected void content of the reference slab on MP4 (outside the wheel path) is steadily decreasing over the depth over the core, the void content in the wheelpath stays more or less the same over the whole depth, indicating an improvement regarding acoustic performance. It is also interesting to see that the reference slabs have in general lower void contents than the optimized mixtures.

Additionally, a rather strong decrease in connected voids is observed for the reference slab SDA 4. In comparison, the optimized mixture M3 retains its connectivity throughout the depth indicating a favorable acoustic property. However, the comparability between the reference plate and the optimized mixture is limited.

#### MMLS3 acoustic absorption

**Fig. 23:**
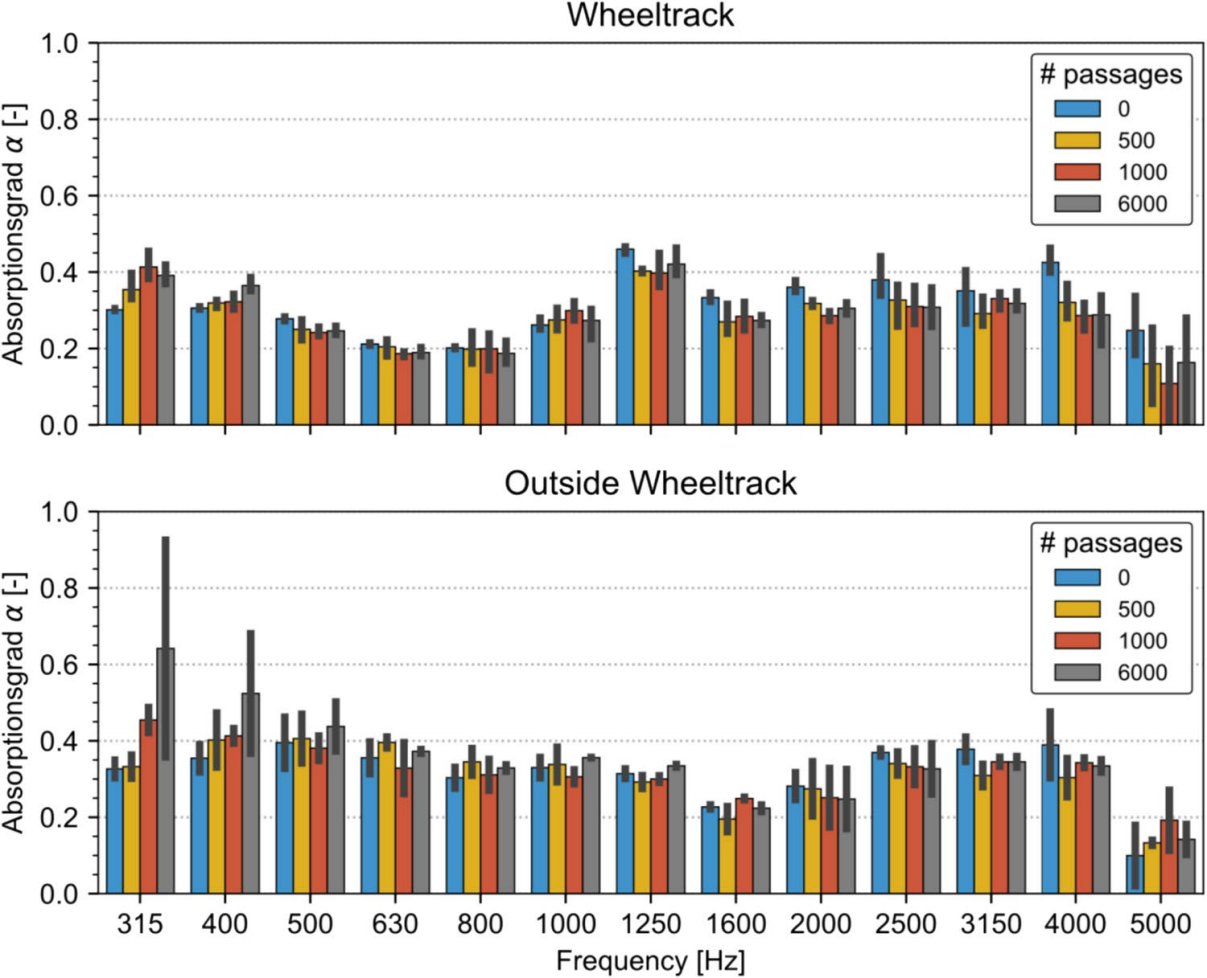
Comparison of sound absorption measurements of the reference plate SDA 4-S for measurements inside (up) and outside wheelpath. (below). Error bars indicate 1 standard deviation

In Fig. 23, the averaged spectral sound absorption measurement is shown for the reference plate SDA 4-S after 0, 500, 1000, and 6000 cycles on the MMLS3. The figure shows that outside the wheel track, not very much has happened in terms of sound absorption between the cycles (except for low frequencies, which might be a measurement artefact).

Regarding the measurement in the wheel track, a slight trend to lower sound absorption is shown between 0 and 500 cycles. However, statistically significant differences (significance level *p* < 0.05) can only be observed for 1250, 2000, and 4000 Hz.

#### MMLS3 air resistivity

**Fig. 24:**
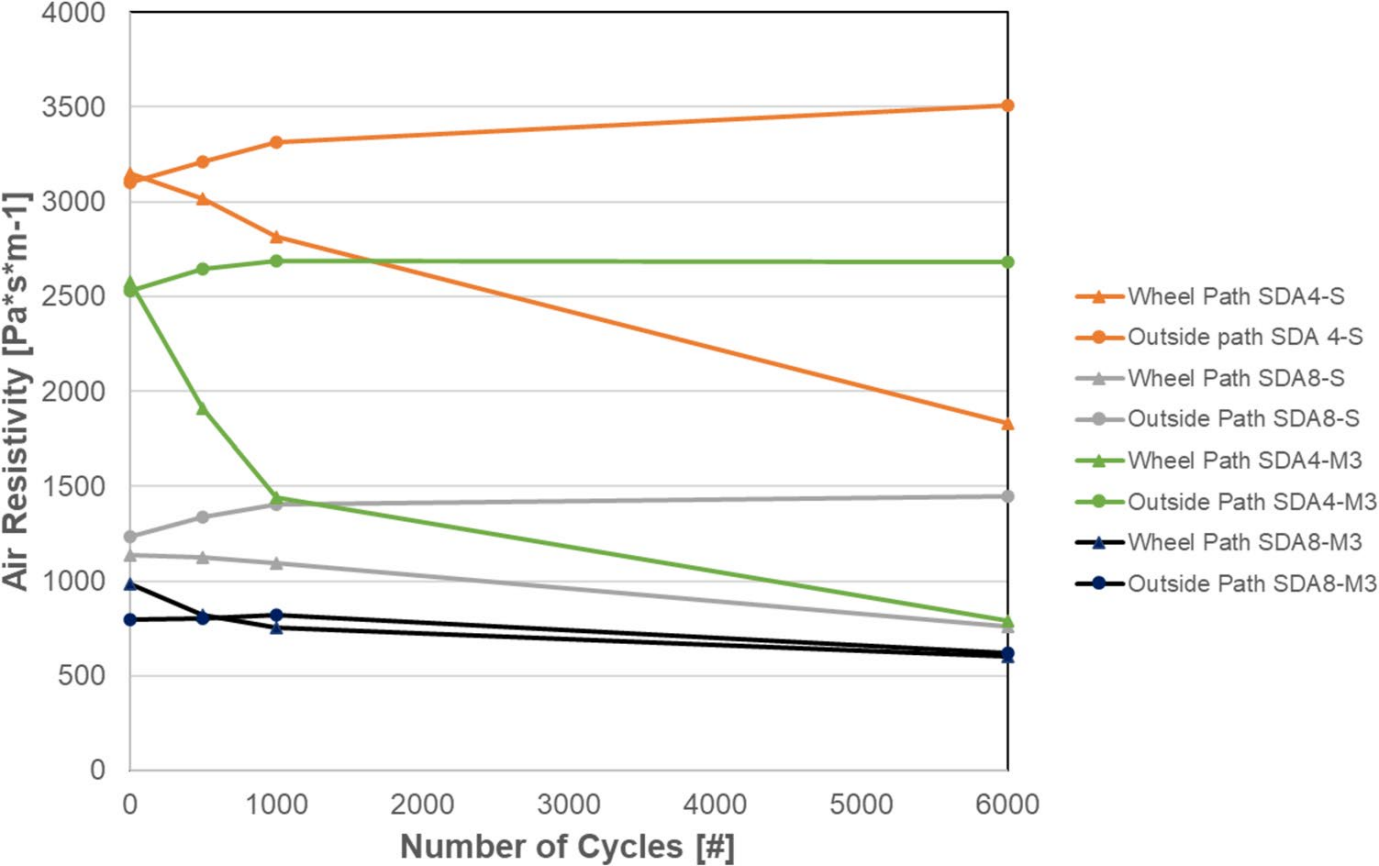
Air resistivity results of MMLS3 slabs for SDA 4 and SDA 8 samples

Air resistivity development in the wheel path and outside the wheel path after exposure to loading cycles is shown in Fig. 24. After 6000 loading cycles, the values have remained constant outside of the wheel path and they have been reduced in the wheel path. This effect has been observed for all four tested slabs. The SDA 4 slabs have higher air resistivity than the SDA 8 slabs and the modified mixtures (M3) have lower air resistivity than the benchmark. The lower air resistivity of SDA 8 is expected as the void size on average is larger due to large aggregates. The values observed corroborate values measured in the field for similar new mixtures (Bühlmann and Hammer 2017). The reduction in the values after exposure to loads is the opposite effect that has been observed in field measurements where the aged pavements have an increased air resistivity (Bühlmann et al. 2017). The cause of the in situ reduction can be primarily attributed to the compaction of the void structure due to traffic. The MMLS sample have shown little compaction as a result of the loading; this is especially the case for the M3 mixtures with the high modulus binder that keeps the structure intact even after the application of 6000 loading cycles. This is also observed with the CT measurements that characterize the void structure after loading and the surface texture showing less of a change in the surface texture. The consistent decrease in air resistivity in the current experiments in the wheel path can be attributed to the fact that due to the rut, the device cannot form a sealed contact with the pavement surface and the air can escape causing a lower air resistivity. Therefore, for the noise modeling in the next chapter, the air resistivity values outside of the wheel path are used.

#### Acoustic simulations of optimized mixtures (m-scale)

The different noise contribution from the measurements of surface texture, absorption, and air resistivity can be compared using the SPERoN-simulation. Simulation results have shown that the loading from the MMLS3 has a direct impact on the simulated noise levels. After 6000 cycles, the average sound emission in the wheel path was simulated to be 85.9 dB compared to 84.1 dB before being exposed to the loading. Thus, after the 6000 cycles, the predicted sound pressure level is increased by 1.8 dB. In brackets are the values shown as a difference from the Swiss noise calculation reference model StL-86 + .

The simulation shows that the initial noise levels for the Reference slab SDA 4 amounts to − 7.5 dB, a very good initial level for SDA 4 road surfaces. After the application of MMLS3, the good acoustic characteristics have decreased by 1.8 dB. On the other hand, for the modified mixture SDA 4 M3, the simulated acoustic noise levels have remained more or less constant between the unloaded measurement points (0) and the loaded measurement points (6000).

When interpreting the results, it must be taken into account that the mechanical response and acoustical aging behavior of the slabs in MMLS3 differs from the behavior in the field. This is because the load in the MMLS3 does exhibit neither any shear forces nor any dirt particles from the environment enter the samples. Therefore, the given acoustical ageing rate of the slabs can only be translated to reality to a limited extent.

**Fig. 25:**
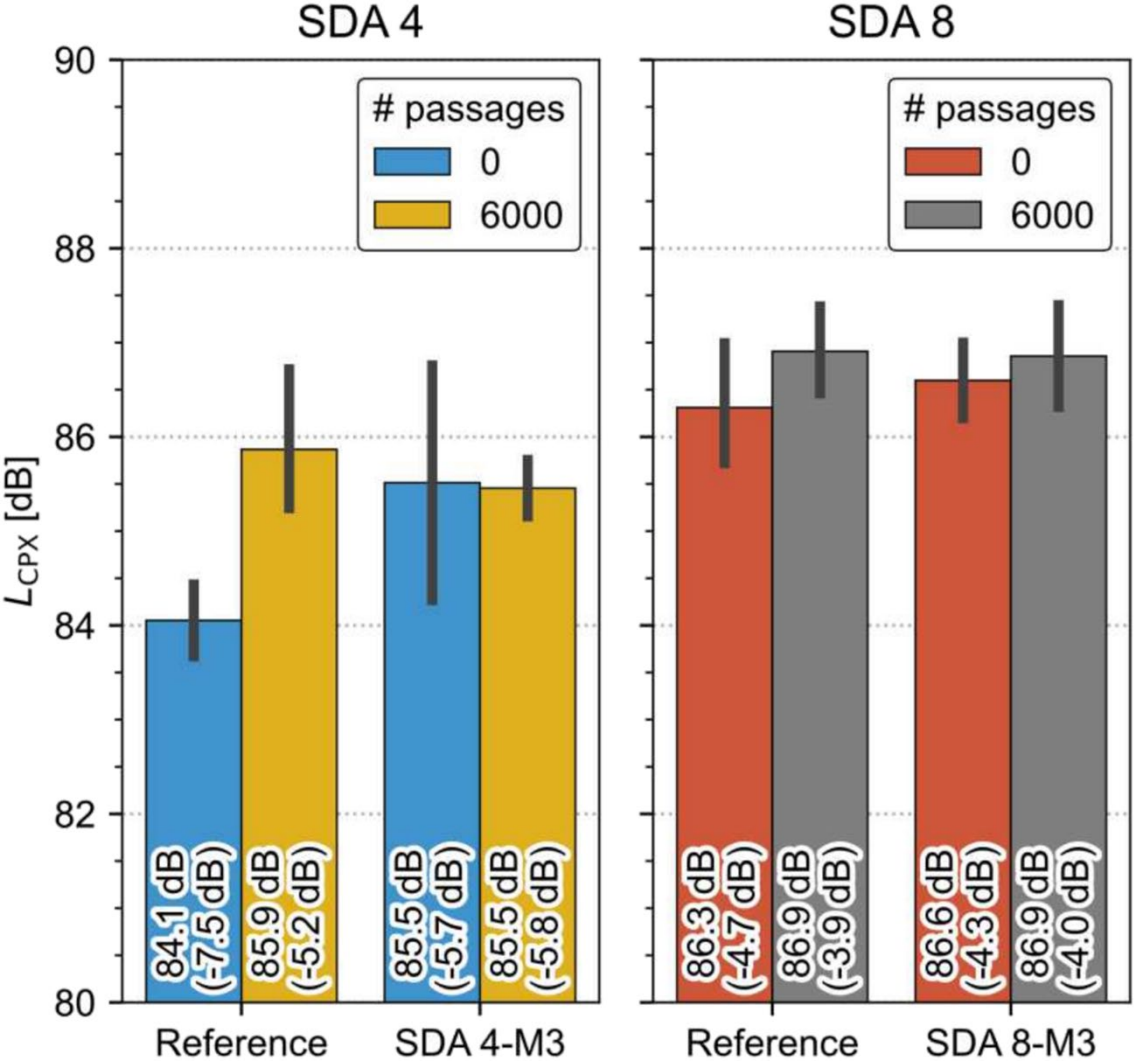
Summary of acoustic results of SDA 4 and SDA 8 for reference and optimized mixtures (M3) unaged and aged with MMLS3

In Fig. 25, the absolute levels for SDA 4 and SDA 8 are shown. This graph illustrates that SDA 4 has the better noise reduction potential initially as well as after the 6000 MMLS3 cycles. It can also be seen that the acoustic performance decreases more for the SDA 4 than for SDA 8. However, this does not mean that it is performing better in the field. The field performance as seen from the measured data has shown a different behavior (see Fig. 1). It has to be stressed that the aging mechanism applied in the lab is more limited compared to the ageing mechanisms experienced in the field. In the field, clogging through dirt ingress is one of the main acoustical aging mechanisms. This was not incorporated in the current study, as a reliable method for simulating dirt ingress is not known. Also, the MMLS3 employs unidirectional loading. In the field, breaking, acceleration, and turning of wheels apply more varied forces on the road surface than the controlled and consistent loading in a lab setting. These variations in loading conditions can significantly impact the aging mechanisms experienced by materials in real-world scenarios. The absence of dirt ingress and the lack of dynamic loading conditions in the lab can indeed result in a different aging profile compared to what is observed in actual field conditions.

A comparison of an aged sample (MP2 after 6000 cycles) and an unaged sample (MP4, 0 cycles) shows a reduction of acoustic performance in most cases shown in Fig. 25. The cause for the increase may be described in the following. It is evident that the vibration noise component is reduced in the upper frequencies (500–2000 Hz). This corresponds well to the observation of reduced texture levels as indicated in the previous section. Furthermore, large differences are visible with the airflow noise. Due to the effects of the loading wheel spreading the bitumen on the surface, smoothing the macrotexture, and blocking part of the surface porosity, air has more difficulty escaping. This leads to increased airflow sound, which is typically observed in the middle to the upper frequency range. The slightly reduced sound absorption observed is another indication for this phenomena.

## Conclusions

A multi-scale approach was implemented to investigate mechanical and acoustic performance of SDA 4, SDA 6, and SDA 8 mixtures from mm- to km-scale. Table 4 shows a summary of the critical results. Benchmark mixtures were developed using the optimum acoustic in situ performance of these mixtures using 200 test sections. As a first step, the mechanical performance of the benchmark mixtures were investigated. It was shown that the overall performance ranking of the benchmark mixtures was as follows: SDA 4 > SDA 6 > SDA 8. As a second step, combinations of binder and filler and additives were used to characterize the mastic. The best performing mastic was used thereafter in producing the modified mixtures. The SDA 4 mixtures were first modified and tested mechanically using a wide variety of tests in order to determine their performance. Thereafter, the optimized modification was applied to SDA 8 slabs. From the experimental results, the following conclusions can be drawn:

- At the mastic scale, it was observed that the filler primarily had a stiffening effect with higher complex modulus and lower phase angle at higher temperatures above 30 °C, with Zeobit filler having a stronger effect.
- At the mastic scale, highly modified binder with limestone and Zeobit filler as well as a combination of Zeobit filler and hydrated lime had the best results that were also similar.
- The optimized modification for the mixtures was a combination of highly modified binder, stiffening filler (Zeobit), and hydrated lime to improve water sensitivity.
- It should be noted that hydrated lime has a relatively high environmental footprint and this aspect should be considered when choosing the optimum materials.
- Laboratory scale mixture performance tests have shown that the modification improved the water sensitivity, fatigue, and cyclic compression resistance and had similar low temperature cracking results in comparison to the benchmark mixture.
- The Micro-Deval-IMP test has shown higher particle loss for SDA 8 mixtures. The modified SDA 4 mixtures showed similar particle loss to the benchmark mixtures.
- The large-scale MMLS tests showed that after 50,000 loading cycles, the modified M3 mixtures showed less rutting in comparison to the reference. This effect was greater for the SDA 8 mixtures. The modified mixtures had similar changes in surface texture and acoustic performance to the benchmark mixtures before and after the wheeltracking.
- Using the MMLS3 load simulator and measurements before and after exposure to loads, the acoustic modeling has shown that the modified mixture SDA 4-M3 and SDA 8-M3 noise levels improved between the unloaded measurement points and the loaded measurement points. This indicates that the modification maintained the structural integrity of the mixture after loading that contribute to better acoustical performance. This was confirmed by the X-ray tomograms of the loaded and unloaded samples showing that the void connectivity of the modified mixtures was larger, contributing to better noise absorption.
- Based on the conducted research and as summarized in Table 4, the mixtures SDA4-M3 and SDA8-M3 are recommended for mechanical and acoustic performance.

**Table 4:**
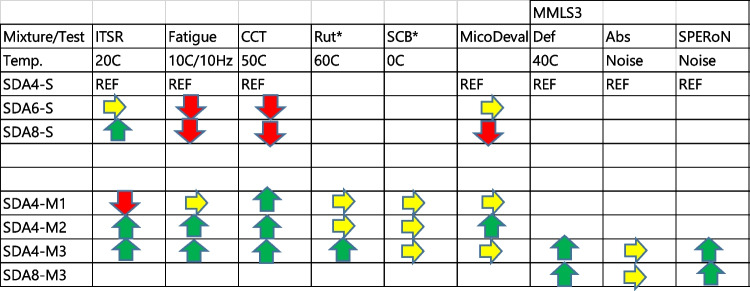
Summary of results: arrows indicate results in comparison to SDA 4-S standard mixture; green arrow = improved performance; yellow arrow = similar performance; red arrow = worst performance. SCB and rutting results are only for the modified mixtures; yellow arrow shows similar results and green arrow shows better performance in comparison to the modified mixtures

## Appendix 1 X-ray tomography measurement and analysis

The tomograph used consists of a high-energy, micro-focus, reflection-based X-ray source (by FineTec, model FOMR 300.03Y RT) and by a two-dimensional flat panel detector (by PerkinElmer, model XRD 1611-CP3) with 4096 × 4096 pixels, each with physical size of 100 μm. Each pixel is made of amorphous Si and is covered by a thin layer of CsI, acting as an X-ray scintillator, converting the respective photons into visible light ones, then detected by the amorphous Si element.

In front of the X-ray source, a 1-mm-thick Cu disk was placed in order to reduce the content of low-energy photons in the X-ray source emission spectrum, thus respective beam hardening artifacts in the final tomogram.

The detector was configured to exploit a 2 × 2 pixel binning, i.e., the average signal from 2 × 2 adjacent pixels was assigned to a virtual pixel with double the size of the physical ones. This led to a downsampling of the pixels’ two-dimensional array by a factor of 2 along each dimension, leading to an actual physical pixel size $p$ = 200 μm.

Each tomography measurement consisted of 2700 radiographs acquired over 360° of specimen rotation around the specimen symmetry axis. At each specimen orientation angle, a respective radiograph was actually the result of averaging five successive radiographs, each acquired within 2 s.

Each averaged radiograph at any given angle was saved as a 2D TIFF image file with a 16-bit depth. The source-to-specimen distance, ${d}_{S-s}$, was equal to 211.58 mm, while the source-to-detector distance, ${d}_{S-d}$, was equal to 1407.57 mm. Because of the cone geometry of the X-ray beam, the radiographs were characterized by a geometrical magnification factor, $M\equiv {d}_{S-d}/{d}_{S-s}$, value of ≅ 6.65, leading to an effective pixel size $\widetilde{p}\equiv {~}^{p}\!\left/ \!{~}_{M}\right.$≅ 0.03 mm. $\widetilde{p}$ is thus also the final size of the isotropic voxels each tomogram consists of. However, it is not equal to the spatial resolution of the tomograms, which is typically lower; i.e., it has a larger numerical value, due to different sources of image noise and response contribution of the whole imaging system. A ball-park value for an upper bound of the actual spatial resolution could be fixed at double the voxel size, i.e., 0.06 mm.

The final tomograms were reconstructed by a GPU processing-based implementation of the Feldkamp-David-Kress cone beam filtered back-projection reconstruction algorithm (Feldkamp et al. 1984), available within the XAct software (Ver. 1.1) by RX-Solutions (https://www.rx-solutions.com/).

## Data Availability

All data used in this study will be available upon request.
